# Supplementary material for: Inadequate sanitation in healthcare facilities: A comprehensive evaluation of toilets in major hospitals in Dhaka, Bangladesh
Source: PLoS One. 2024 May 22;19(5):e0295879. doi: 10.1371/journal.pone.0295879 (PMC11111017; doi:10.1371/journal.pone.0295879)

**Inadequate Sanitation in Healthcare Facilities: A Comprehensive Evaluation of Toilets in Major Hospitals in Dhaka, Bangladesh**

**Nuhu Amin^1,2*^,** Tim Foster^1^, Md. Imam Hossain**^2^**, Md Rezaul Hasan**^2^**, Supriya Sarkar^3^, Aninda Rahman^4^, [Shaikh Daud Adnan](https://pubmed.ncbi.nlm.nih.gov/?term=Adnan+SD&cauthor_id=36623655)^3^, Mahbubur Rahman**^2^**, Juliet Willetts^1^

^1^Institute for Sustainable Futures, University of Technology Sydney, 235 Jones St, Ultimo, NSW, 2007, Australia

^2^Environmental Health and WASH, Health System and Population Studies Division, International Centre for Diarrhoeal Disease Research, Bangladesh (icddr,b), Dhaka, Bangladesh

^3^Hospital Services Management, Directorate General of Health Services (DGHS), MoH&FW, Mohakhali, Dhaka-1212, Bangladesh

^4^Communicable Disease Control (CDC) Program, Directorate General of Health Services (DGHS), MoH&FW, Mohakhali, Dhaka-1212, Bangladesh

*Correspond to [nuhu.amin@icddrb.org](mailto:nuhu.amin@icddrb.org)

S1 Table: Users to toilet ratio in 10 government hospitals and two non-government hospitals in Dhaka city, considering only functional toilets

|  | Users-to-toilet ratio | | | | | |
| --- | --- | --- | --- | --- | --- | --- |
| Type of hospital (# of toilets) | Inpatient departments | | | Outpatient departments | | |
|  | Toilet : patients^*^ | Toilet : staff^†^ | Toilet : mix^‡^ | Toilet : patients | Toilet : staff | Toilet : mix |
| **Government hospitals (n=1562)** | **28:1** | **16:1** | **36:1** | **321:1** | **31:1** | **246:1** |
| **General hospitals (n=354)** | **9:1** | **14:1** | **66:1** | **284:1** | **17:1** | **159:1** |
| H1(n=88) | 6:1 | 49:1 | 77:1 | 0 | 16:1 | 72:1 |
| H2 (n=266) | 10:1 | 6:1 | 57:1 | 284:1 | 19:1 | 195:1 |
| **Medical College hospitals (469)** | **9:1** | **13:1** | **22:1** | **406:1** | **96:1** | **425:1** |
| H3 (n=182) | 50:1 | 17:1 | 45:1 | 175:1 | 18:1 | 358:1 |
| H4 (n=287) | 15:1 | 10:1 | 14:1 | 498:1 | 134:1 | 486:1 |
| **Specialized hospitals (n=739)** | **34:1** | **18:1** | **31:1** | **343:1** | **12:1** | **202:1** |
| ***Treat mainly infectious diseases*** |  |  |  |  |  |  |
| H5 (n=31) | 14:1 | 13:1 | 0 | 450:1 | 3:1 | 0 |
| H6 (n=122) | 18:1 | 8:1 | 194:1 | 74:1 | 6:1 | 0 |
| ***Treat mainly chronic diseases*** |  |  |  |  |  |  |
| H7 (n=199) | 52:1 | 10:1 | 31:1 | 0 | 27:1 | 939:1 |
| H8 (n=119) | 62:1 | 80:1 | 31:1 | 370:1 | 10:1 | 35:1 |
| H9 (n=152) | 16:1 | 18:1 | 41:1 | 264:1 | 25:1 | 48:1 |
| H10 (n=116) | 68:1 | 7:1 | 25:1 | 101:1 | 3:1 | 368:1 |
| **Private hospitals (n=134)** | **9:1** | **14:1** | **18:1** | **115:1** | **32:1** | **14:1** |
| P1 (n=83) | 11:1 | 12:1 | 13:1 | 259:1 | 37:1 | 30:1 |
| P2 (n=51) | 4:1 | 19:1 | 23:1 | 0 | 5:1 | 4:1 |
| **Overall total (n=1696)** | **26:1** | **16:1** | **31:1** | **302:1** | **31:1** | **232:1** |

^*^Patients users: Patients including caregivers of hospitals reported by hospital authority during the day of data collection

^†^Staff: toilet user’s included doctors, nurse, and cleaning staff

^‡^Mix user: The toilet used by both patients, caregivers and staff

Figure 2: perceived cleanliness scale of the data collectors

S1 Fig: Cleanliness scale of the toilets used by the data collectors.


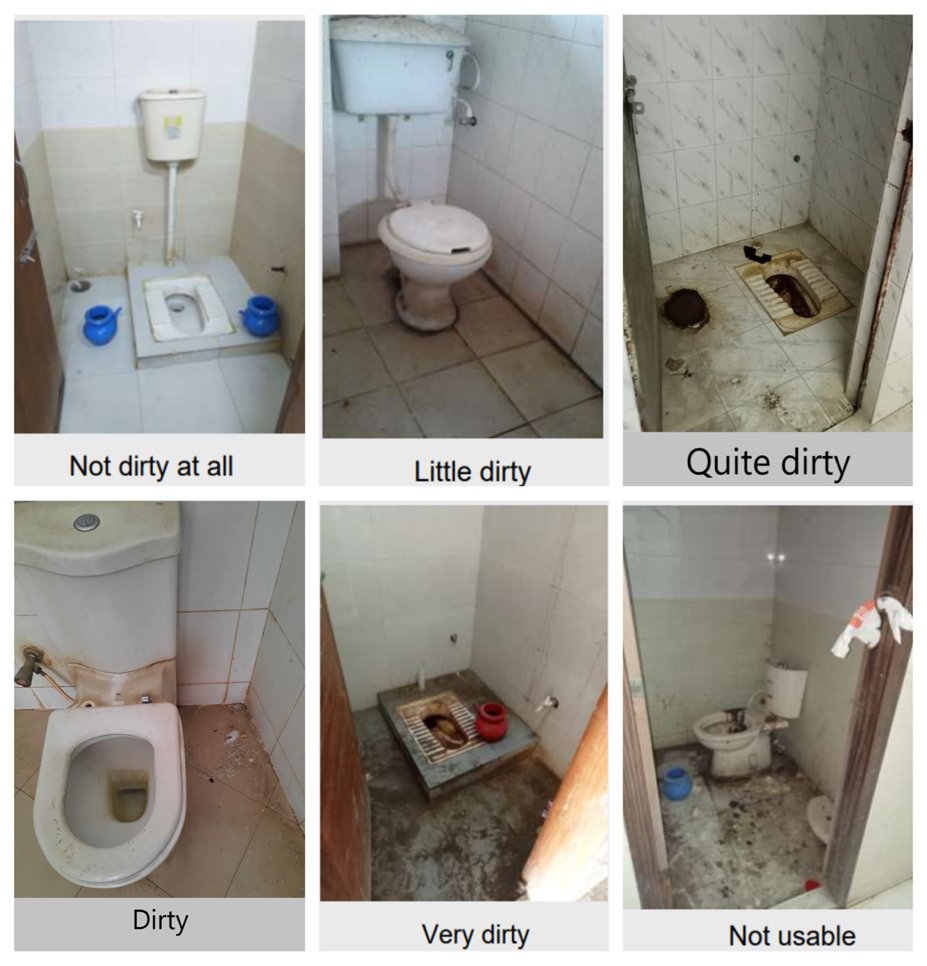

Supplement: S1 File — (DOCX) [file pone.0295879.s001.docx]
