# Supplementary material for: Inadequate sanitation in healthcare facilities: A comprehensive evaluation of toilets in major hospitals in Dhaka, Bangladesh
Source: PLoS One. 2024 May 22;19(5):e0295879. doi: 10.1371/journal.pone.0295879 (PMC11111017; doi:10.1371/journal.pone.0295879)
Supplement: Attachment — Submitted filename: PONE-D-23-39815_reviewed.pdf [file pone.0295879.s002.pdf]

PLOS ONE

Inadequate Sanitation in Healthcare Facilities: A Comprehensive Evaluation of Toilets in Major Hospitals in Dhaka, Bangladesh

--Manuscript Draft--

**Manuscript Number:**	PONE-D-23-39815
**Article Type:**	Research Article
**Full Title:**	Inadequate Sanitation in Healthcare Facilities: A Comprehensive Evaluation of Toilets in Major Hospitals in Dhaka, Bangladesh
**Short Title:**	Inadequate Sanitation in Healthcare Facilities: Evaluation of Toilets in Major Hospitals in Dhaka, Bangladesh
**Corresponding Author:**	Nuhu Amin<br>ICDDRB: International Centre for Diarrhoeal Disease Research Bangladesh<br>Dhaka, 01 BANGLADESH
**Keywords:**	Healthcare facility; sanitation; Toilet functionality and cleanliness; Toilet-to-users ratio; Infection control and prevention; SDG6
**Abstract:**	Background<br><br>Lack of access to functional and hygienic toilets in healthcare facilities (HCFs) is a significant public health issue in low- and middle-income countries (LMICs), leading to the transmission of infectious diseases. Globally, there is a lack of studies estimating toilet conditions and appropriate toilet-to-user ratios in large urban hospitals in LMICs. We conducted a cross-sectional study in 10-government and two-private hospitals to explore the availability, functionality, cleanliness, and toilet-to-users ratio in Dhaka, Bangladesh.<br><br>Methods<br><br>From Aug-Dec 2022, we undertook infrastructure assessments of toilets in selected hospitals. We observed all toilets and recorded attributes of intended users, including sex, disability status, patient status (in-patient/out-patient/caregiver) and/or staff (doctor/nurse/cleaner/mixed). Toilet functionality was defined according to criteria used by the WHO/UNICEF Joint-Monitoring Programme in HCFs. Toilet cleanliness was assessed, considering visible feces on any surface, strong fecal odor, presence of flies, sputum, insects, and rodents, and solid waste.<br><br>Results<br><br>Amongst 2875 toilets, 2459 (86%) were observed. Sixty-eight-percent of government hospital toilets and 92% of private hospital toilets were functional. Only 33% of toilets in government hospitals and 56% in private hospitals were clean. A high toilet-to-users ratio was observed in government hospitals' outpatients service (1:214) compared to inpatients service (1:17). Toilet-to-users ratio was also high in private hospitals' outpatients service (1:94) compared to inpatients wards (1:9). Only 3% toilets had bins for menstrual-pad disposal and <1% of toilets had facilities for disabled people.<br><br>Conclusion<br><br>A high percentage of unclean toilets coupled with high toilet-to-users ratio hinders the achievement of SDG by 2030 and risks poor infection-control. Increasing the number of usable, clean toilets in proportion to users is crucial. The findings suggest an urgent call for attention to ensure basic sanitation facilities in Dhaka's HCFs. This necessitates allocating resources for adequate toilets, maintenance staff, cleanliness, along with strong leadership.
**Order of Authors:**	Nuhu Amin
	Tim Foster
	Md Imam Hossain

Powered by Editorial Manager® and ProduXion Manager® from Aries Systems Corporation

	Md Rezaul Hasan
	Supriya Sarkar
	Aninda Rahman
	Shaikh Daud Adnan
	Mahbubur Rahman
	Juliet Willetts
**Additional Information:**	
**Question**	**Response**
**Financial Disclosure**<br><br>Enter a financial disclosure statement that describes the sources of funding for the work included in this submission. Review the submission guidelines for detailed requirements. View published research articles from *PLOS ONE* for specific examples.<br><br>This statement is required for submission and **will appear in the published article** if the submission is accepted. Please make sure it is accurate.<br><br>**Funded studies**<br>Enter a statement with the following details:<br>• Initials of the authors who received each award<br>• Grant numbers awarded to each author<br>• The full name of each funder<br>• URL of each funder website<br>• Did the sponsors or funders play any role in the study design, data collection and analysis, decision to publish, or preparation of the manuscript?<br><br>Did you receive funding for this work?	Yes
Please add funding details.<br>as follow-up to "**Financial Disclosure**<br><br>Enter a financial disclosure statement that describes the sources of funding for the work included in this submission. Review the submission guidelines for detailed requirements. View published research articles from *PLOS ONE* for specific examples.	This project has been funded by the Bill & Melinda Gates foundation (BMGF) [grant number INV-045360].

This statement is required for submission and **will appear in the published article** if the submission is accepted. Please make sure it is accurate.<br><br>**Funded studies**<br>Enter a statement with the following details:<br>• Initials of the authors who received each award<br>• Grant numbers awarded to each author<br>• The full name of each funder<br>• URL of each funder website<br>• Did the sponsors or funders play any role in the study design, data collection and analysis, decision to publish, or preparation of the manuscript?<br><br>Did you receive funding for this work?"	
Please select the country of your main research funder (please select carefully as in some cases this is used in fee calculation).<br>as follow-up to "**Financial Disclosure**<br><br>Enter a financial disclosure statement that describes the sources of funding for the work included in this submission. Review the submission guidelines for detailed requirements. View published research articles from *PLOS ONE* for specific examples.<br><br>This statement is required for submission and **will appear in the published article** if the submission is accepted. Please make sure it is accurate.	UNITED STATES - US

**Funded studies**<br>Enter a statement with the following details:<br>• Initials of the authors who received each award<br>• Grant numbers awarded to each author<br>• The full name of each funder<br>• URL of each funder website<br>• Did the sponsors or funders play any role in the study design, data collection and analysis, decision to publish, or preparation of the manuscript?<br><br>Did you receive funding for this work?"	
**Competing Interests**<br><br>Use the instructions below to enter a competing interest statement for this submission. On behalf of all authors, disclose any competing interests that could be perceived to bias this work—acknowledging all financial support and any other relevant financial or non-financial competing interests.<br><br>This statement is required for submission and **will appear in the published article** if the submission is accepted. Please make sure it is accurate and that any funding sources listed in your Funding Information later in the submission form are also declared in your Financial Disclosure statement.<br><br>View published research articles from *PLOS ONE* for specific examples.	The authors have declared that no competing interests exist.

**NO authors have competing interests**<br><br>Enter: *The authors have declared that no competing interests exist.*<br><br>**Authors with competing interests**<br><br>Enter competing interest details beginning with this statement:<br><br>*I have read the journal's policy and the authors of this manuscript have the following competing interests: [insert competing interests here]*<br><br>* typeset	
**Ethics Statement**<br><br>Enter an ethics statement for this submission. This statement is required if the study involved:<br><br>• Human participants<br>• Human specimens or tissue<br>• Vertebrate animals or cephalopods<br>• Vertebrate embryos or tissues<br>• Field research<br><br>Write "N/A" if the submission does not require an ethics statement.<br><br>General guidance is provided below. Consult the submission guidelines for detailed instructions. **Make sure that all information entered here is included in the Methods section of the manuscript.**	We obtained written informed consent from all participants in the study and received written approvals from the director general of health service (DGHS). We also obtained formal written approvals from all hospital directors to carry out the interviews. The study protocol received approval from both the ethical review committee of icddr,b, Dhaka, Bangladesh and the institutional review board of University of Technology Sydney, Australia.

Powered by Editorial Manager® and ProduXion Manager® from Aries Systems Corporation

**Format for specific study types**<br><br>**Human Subject Research (involving human participants and/or tissue)**<br>• Give the name of the institutional review board or ethics committee that approved the study<br>• Include the approval number and/or a statement indicating approval of this research<br>• Indicate the form of consent obtained (written/oral) or the reason that consent was not obtained (e.g. the data were analyzed anonymously)<br><br>**Animal Research (involving vertebrate animals, embryos or tissues)**<br>• Provide the name of the Institutional Animal Care and Use Committee (IACUC) or other relevant ethics board that reviewed the study protocol, and indicate whether they approved this research or granted a formal waiver of ethical approval<br>• Include an approval number if one was obtained<br>• If the study involved *non-human primates*, add *additional details* about animal welfare and steps taken to ameliorate suffering<br>• If anesthesia, euthanasia, or any kind of animal sacrifice is part of the study, include briefly which substances and/or methods were applied<br><br>**Field Research**<br><br>Include the following details if this study involves the collection of plant, animal, or other materials from a natural setting:<br>• Field permit number<br>• Name of the institution or relevant body that granted permission	
**Data Availability**<br><br>Authors are required to make all data underlying the findings described fully available, without restriction, and from the time of publication. PLOS allows rare exceptions to address legal and ethical concerns. See the PLOS Data Policy and FAQ for detailed information.	Yes - all data are fully available without restriction

A Data Availability Statement describing where the data can be found is required at submission. Your answers to this question constitute the Data Availability Statement and **will be published in the article**, if accepted.<br><br>**Important:** Stating 'data available on request from the author' is not sufficient. If your data are only available upon request, select 'No' for the first question and explain your exceptional situation in the text box.<br><br>Do the authors confirm that all data underlying the findings described in their manuscript are fully available without restriction?	
**Describe where the data may be found in full sentences. If you are copying our sample text, replace any instances of XXX with the appropriate details.**<br><br>• If the data are **held or will be held in a public repository**, include URLs, accession numbers or DOIs. If this information will only be available after acceptance, indicate this by ticking the box below. For example: *All XXX files are available from the XXX database (accession number(s) XXX, XXX.*).<br>• If the data are all contained **within the manuscript and/or Supporting Information files**, enter the following: *All relevant data are within the manuscript and its Supporting Information files.*<br>• If neither of these applies but you are able to provide **details of access elsewhere**, with or without limitations, please do so. For example:<br><br>*Data cannot be shared publicly because of [XXX]. Data are available from the XXX Institutional Data Access / Ethics Committee (contact via XXX) for researchers who meet the criteria for access to confidential data.*<br><br>*The data underlying the results presented in the study are available from (include the name of the third party*	All relevant data are within the paper and its Supporting Information files.

and contact information or URL).<br>• This text is appropriate if the data are owned by a third party and authors do not have permission to share the data.<br><br>* typeset	
Additional data availability information:	

Manuscript body Click here to access/download;Manuscript;Manuscript.docx

Inadequate Sanitation in Healthcare Facilities: A Comprehensive Evaluation of Toilets in Major Hospitals in Dhaka, Bangladesh

Nuhu Amin[1,2*], Tim Foster[1], Md. Imam Hossain[2], Md Rezaul Hasan[2], Supriya Sarkar[3], Aninda Rahman[4], Shaikh Daud Adnan[3] Mahbubur Rahman[2], Juliet Willetts[1]

[1]Institute for Sustainable Futures, University of Technology Sydney, 235 Jones St, Ultimo, NSW, 2007, Australia
[2]Environmental Health and WASH, Health System and Population Studies Division, International Centre for Diarrhoeal Disease Research, Bangladesh (icddr,b), Dhaka, Bangladesh
[3]Hospital Services Management, Directorate General of Health Services (DGHS), MoH&FW, Mohakhali, Dhaka-1212, Bangladesh
[4]Communicable Disease Control (CDC) Program, Directorate General of Health Services (DGHS), MoH&FW, Mohakhali, Dhaka-1212, Bangladesh

*Correspond to mdnuhu.amin@student.uts.edu

Keywords: Healthcare facility; Sanitation; Toilet functionality and cleanliness; Toilet-to-users ratio; Infection control and prevention; SDG6

ABSTRACT

Background: Lack of access to functional and hygienic toilets in healthcare facilities (HCFs) is a significant public health issue in low- and middle-income countries (LMICs), leading to the transmission of infectious diseases. Globally, there is a lack of studies estimating toilet conditions and appropriate toilet-to-user ratios in large urban hospitals in LMICs. We conducted a cross-sectional study in 10-government and two-private hospitals to explore the availability, functionality, cleanliness, and toilet-to-users ratio in Dhaka, Bangladesh.

Methods: From Aug-Dec 2022, we undertook infrastructure assessments of toilets in selected hospitals. We observed all toilets and recorded attributes of intended users, including sex, disability status, patient status (in-patient/out-patient/caregiver) and/or staff (doctor/nurse/cleaner/mixed). Toilet functionality was defined according to criteria used by the WHO/UNICEF Joint-Monitoring Programme in HCFs. Toilet cleanliness was assessed, considering visible feces on any surface, strong fecal odor, presence of flies, sputum, insects, and rodents, and solid waste.

Results: Amongst 2875 toilets, 2459 (86%) were observed. Sixty-eight-percent of government hospital toilets and 92% of private hospital toilets were functional. Only 33% of toilets in government hospitals and 56% in private hospitals were clean. A high toilet-to-users ratio was observed in government hospitals' outpatients service (1:214) compared to inpatients service (1:17). Toilet-to-users ratio was also high in private hospitals' outpatients service (1:94) compared to inpatients wards (1:9). Only 3% toilets had bins for menstrual-pad disposal and <1% of toilets had facilities for disabled people.

Conclusion: A high percentage of unclean toilets coupled with high toilet-to-users ratio hinders the achievement of SDG by 2030 and risks poor infection-control. Increasing the number of usable, clean toilets in proportion to users is crucial. The findings suggest an urgent call for attention to ensure basic sanitation facilities in Dhaka's HCFs. This necessitates allocating resources for adequate toilets, maintenance staff, cleanliness, along with strong leadership.

Introduction

Globally, approximately 10% of healthcare facilities (HCF) have no sanitation services, meaning approximately 800 million people seek healthcare at facilities without toilets [1]. This problem is even more significant in low- and middle-income countries (LMICs), where 50% of HCFs lack reliable water services and only 21% of HCFs have basic sanitation services [2]. Addressing this issue is a priority for these countries to achieve sustainable Development Goals (SDGs) by 2030 [1]. Furthermore, the availability and effectiveness of such services in HCFs is critically important given the type of healthcare services provided by HCFs. The absence of adequate water, sanitation and hygiene (WASH) services significantly contributes to substandard healthcare, influencing infection rates, service utilization, staff productivity, and the dignity of patients and healthcare workers [3]. Several studies suggest that the enhancement of WASH in HCFs could potentially prevent millions of deaths and lead to substantial financial savings in the order of billions of dollars [3, 4].

Toilet availability, functionality and cleanliness play a vital role in achieving safe sanitation services in HCFs [5]. A recent global report on WASH in HCFs by the WHO-UNICEF Joint Monitoring Programme (JMP) highlights that although many HCFs have toilets on their premises, a significant number of them are non-functional and unusable [6]. In 2021, the JMP also reported that 20% of HCFs in LMICs had no sanitation service, and 63% of toilets in LMICs did not meet basic sanitation standards, indicating the presence of unimproved and non-functional sanitation facilities [7]. Recent estimates also suggested that globally, only 69 countries had estimates for coverage of improved sanitation facilities in HCFs [7]. However, there is a global lack of data pertaining to toilet functionality and cleanliness, especially in the large cities in LMICs [2].

Toilet hygiene is important in the control of both enteric and air-borne pathogens- associated illness both in HCFs and the community [8]. Several studies suggest that there have been reported outbreaks indicating the toilet as a source of infection in HCFs for enteric pathogens [9-11]. Numerous enteric pathogens are found in high concentration in stools and therefore in toilets after defecation, particularly during disease outbreak [8]. Unhygienic and dirty toilets pose significant concerns from both a public health and Infection Prevention and Control (IPC) perspective. Unhygienic and dirty toilets serve as breeding grounds for pathogens, including bacteria, viruses, and parasites, which can spread through contact with contaminated surfaces [9, 10, 12, 13]. This risk is high in HCFs, where unclean hospital environment including toilets contribute to healthcare-associated infections, particularly affecting immunocompromised patients [14]. Beyond health risks, unclean toilets compromise satisfaction, dignity and well-being of both patients and staff, notably in healthcare settings where recovery is vital [3]. Maintaining proper IPC measures demands clean environments to prevent transmission, a challenge unclean toilet hinder [14, 15].

Bangladesh stands as one of the world's densely populated countries (>1119 people per square kilometer), with about 22% of people living below the poverty line [16]. The healthcare system in Bangladesh lacks reliability, responsiveness, and accountability, and its shortcomings in delivering healthcare to the public have been demonstrated on numerous occasions [17, 18]. Although medical facilities are concentrated in urban areas, the high population density in mega cities like Dhaka (population density: >23,000 people/km^2) exacerbates the challenges of managing healthcare services [19]. High patient flow could impact toilet functionality in healthcare facilities [20, 21]. However, there is a notable research gap concerning toilet-to-users ratio, particularly in HCFs within LMICs. A recent study conducted in six government hospitals in Dhaka during the COVID-19 pandemic revealed that the overall toilet-to-users ratio was high, at 1:25 in general hospitals [20], compared to the Bangladeshi national WASH standard [22]. The study faced limitations as it could only observe 16% (142/230) of the toilets due to COVID-19 entry restrictions. Furthermore, the study did not evaluate cleanliness in connection with the toilet-to-users ratio, and it also did not include private hospitals to analyze disparities in sanitation services.

The burden of inadequate sanitation facilities in healthcare facilities also disproportionately affects women and individuals with limited mobility [23, 24]. Women constitute over 70% of the health workforce [25], and they are also the primary users of healthcare services, particularly in primary care settings. Regrettably, sanitation services for women, children, and those with mobility challenges are often insufficient or lacking. Ensuring equitable access to fundamental sanitation services and practices constitutes a concrete step toward promoting gender equality [26] and upholding the universal human rights to water, sanitation, and health. Although facilities such as gender-segregated and hygienic toilets with waste disposal bins, as well as adequate washing facilities, are crucial for menstruating women, there is a lack of global evidence concerning these parameters in healthcare facilities [1].

There is a notable lack of global data on toilet functionality and hygiene, especially in LMICs such as Bangladesh. In Bangladesh, recent sanitation evaluations have focused on sub-district level hospitals, neglecting larger HCFs in major cities. To address these knowledge gaps in HCFs, we conducted a cross-sectional study to comprehensively explore the availability, functionality, cleanliness, toilet-to-user ratios, and adequacy of sanitation facilities in 10 government and two private hospitals in Dhaka, Bangladesh. We also assessed toilet availability for women, staff, and individuals with limited mobility in all selected hospitals in Dhaka city to determine the number of hospitals with basic sanitation facilities that met their needs. The study focused on addressing existing research gaps and providing insights to enhance sanitation services and infection prevention in a high-density urban context in Bangladesh, with implications for other similar LMIC settings.

Materials and Methods

Study design

We conducted a cross-sectional study between June and December 2022 (post-monsoon to winter season) in 10 government hospitals and two private hospitals. During this period, we conducted structured observations to assess the functionality and cleanliness of the toilets and estimate the number of users for each toilet (i.e., toilet-to-users ratio) in the selected hospitals. A structured observational checklist was used to assess the toilet functionality and cleanliness of the selected hospitals [23].

Enrolment of study hospitals

Before proceeding with the selection of study sites, we prioritized stakeholder engagement by conducting a meeting involving key stakeholders such as the Directorate General of Health Services (DGHS), Dhaka Water Supply and Sewerage Authority (DWASA), policymakers, and national and international NGOs. We revised our hospital selection criteria and data collection tools by incorporating suggestions from the stakeholder meeting. The selection criteria included assessing hospital water and sanitation facilities, year of construction (before or after 2000), hospital type (general, specialized, or medical college and hospitals), and hospital size (small, medium, or large based on the number of beds). Additionally, we considered varied geographic locations within the Dhaka South City Corporation (DSCC) and Dhaka North City Corporation (DNCC) areas. In total, ten government hospitals were selected based on above criteria. This included two general hospitals, two medical college hospitals, and six specialized hospitals. For comparison purposes, we purposively selected two private medical college hospitals (Fig 1) to establish a reference group alongside the government hospitals.

Fig 1: Study flow chart

Selection of toilets

The hospitals in Dhaka city exhibit a wide range of diversity, including variations in size (ranging from 200 beds to 2300 beds), building structures, and patient services, which are not consistent across the hospitals. The usage of sanitation facilities, in terms of both the number

of users and the types of users, also differs across hospitals. For instance, some toilets consist of a single cabin shared by 2-3 users, while outpatient toilets may be used by over 100 users [20]. Considering the complexity and the potential for selection bias, the study undertook a census of all toilets in each hospital except for toilets located in the administrative buildings and toilets exclusively used by students or academic professionals. The benefit of using the above sampling approach for including all toilets in hospitals lies in its ability to provide a comprehensive and representative assessment of sanitation facilities, avoid selection bias, allowing for more accurate analyses and meaningful insights.

Formation of latrine blocks in each hospital floor

After obtaining written approval from the hospital director, trained fieldworkers approached the head nurse of each floor/ward, who were responsible for overseeing the toilets and had a clear understanding of the number of toilets, patients admitted, and caregivers present in their respective ward or duty area on the day of data collection. The objective of the study was explained to the nurses during this interaction. Following discussions with the nurse, one of the investigators (NA) listed total number of toilets present in each floor and divided them into multiple toilet blocks of every hospital. The formation of each block was based on various factors such as the type of users (patients, staff, or both), patient services (inpatients, outpatients, and emergency), all wards (medicine, surgery, gynae, special care, etc.), and gender (male, female, or both).

For instance, if a latrine block was located near the medicine ward, it was designated as the medicine ward toilet block. If there were two separate male and female toilet blocks for patients in one ward, they were considered as two distinct toilet blocks (referred to as block-1 and block-2). Additionally, each cabin within the ward/floor was treated as a separate block due to different users in each cabin. For example, if there were 20 cabins on one floor, the blocks were numbered as latrine block 1-20. Furthermore, staff toilets were also categorized as separate blocks. For example, if a floor had five doctors' toilets, one nurse toilet, and one toilet for other staff members, a total of seven toilet blocks

would be counted. The rationale behind dividing the toilets into multiple blocks on each floor was to ensure the collection of precise information on the number of users (patients, caregivers, and staff) from each specific block.

Structured observation of the toilets

After finalizing the toilet blocks, the fieldworkers administered a structured observational checklist to collect information on the following variables: the number of toilets in each block, the number of patients admitted to the inpatient service, the average number of caregivers visiting for patient care, and the number of staff members (doctors, nurses, ward boys, and cleaners) present during the day of data collection. Similar data was also collected for the outpatient service, laboratory, diagnostics, and special care units such as the operation theatre and intensive care units.

During structured observation, each toilet was assigned a unique identification number. The fieldworkers utilized the Kobo ToolBox software (https://www.kobotoolbox.org/) to conduct the structured observation. The observations encompassed several aspects, including access to the toilet (open or locked), the functionality of the pan or commode (water seal present/absent), the condition of the door (broken or not broken), availability of water, presence of sewage pipe leaks, and overflow of the pan [27]. Additionally, toilet cleanliness was assessed, considering visible feces on any surface of the toilet (including the commode/pan, walls, and floor), strong fecal odor, presence of sputum or saliva on any surface, the existence of flies, cockroaches, insects, and rodents, as well as the presence of solid waste and spider webs. The fieldworkers also evaluated toilet cleanliness based on their own perceived cleanliness during data collection, utilizing a six-point scale ranging from "not dirty at all" to "not usable" (Figure S1) [28]. Furthermore, the fieldworkers explored the types of toilets based on users, including person with limited mobility [29], toilets with menstrual hygiene management (MHM) facility, patient users (male, female, and unisex toilets), healthcare professionals (male, female, and unisex users), and other users such as administrative and support staff. The definition of toilets for person with limited mobility was adapted from Talib *et al* 2016

[29] and MHM facility of toilets were defined as presence of solid disposal bin in inside the toilet [30].

Data analysis

Stata 15.0 (StataCorp LLC, College Station, Texas, USA) and Microsoft Excel 2019 were used for statistical analysis and graph preparation. To determine whether the data was normally distributed, Shapiro-Wilk, Shapiro-Francia, and Skewness and Kurtosis tests were used. To visualize the data, descriptive statistics were performed on the collected variables. Frequency and proportion were calculated for categorical variables such as toilet functionality (functional vs. non-functional), toilet cleanliness (clean vs. not clean), and toilet-to-users ratio. To calculate toilet-to-user ratio between inpatient and outpatient service, we considered the exact toilets user numbers on the day of data collection. We did not compare the toilet-to-users ratio between inpatient and outpatient services because outpatients may only be using the HCF for a few hours to a maximum of half a day, whereas inpatients would typically stay at the HCF for a full day or more. Mean and standard deviation were calculated to estimate the toilet functionality and cleanliness. Two sample Z test of proportions was used to determine whether the toilet functionality and cleanliness differ significantly between inpatient and outpatient services. The outcome and other relevant variables were analyzed and presented based on exposure variables. Descriptive statistics were also conducted separately for four population groups: doctors, nurses/other staff, patients/caregivers, and other latrine users. Univariate and multivariate logistic regression models were performed to evaluate the effects of multiple factors on the toilet functionality and cleanliness. Hosmer–Lemeshow goodness-of-fit test was used to assess how well the model is fit for the analysed data. Variance Inflation Factor (VIF) was performed to test the multicollinearity among the explanatory variables used in the model. The association between toilet functionality, cleanliness and hospital type, hospital year construction, user type, patient's service, gender, user number per toilet was investigated using the univariate analysis. In multivariate analysis, only the exposure variables were used that were found to be statistically

significant in univariate analysis [31]. The multivariate model was adjusted for co-variates such as user type, gender, user number per toilet to overcome the confounding effects. The model was also adjusted with robust standard error to control the clustering effect within hospitals. P-value < 0.05 was considered statistically significant.

Ethical considerations

We obtained written informed consent from all participants in the study and received written approvals from the director general of health service (DGHS). We also obtained formal written approvals from all hospital directors to carry out the interviews. The study protocol received approval from both the ethical review committee of icddr,b, Dhaka, Bangladesh and the institutional review board of University of Technology Sydney, Australia.

Results

Among the 12 selected hospitals, there were two government general hospitals, two government medical college hospitals, six specialized hospitals, and two private hospitals. Out of the selected hospitals, six were established before the year 2000. The number of beds ranged from 250 to 850, with an average of 422 beds per hospital. The highest number of observed toilets was in a 500-bed government medical college hospital (H4=430 toilets), while the lowest number of toilets was found in a 300-bed government specialized hospital (H5=43 toilets). Both private hospitals were 500-bedded, with 86 toilets in hospital P1 and 59 toilets in hospital P2. The total number of toilet users (staff, patients, caregivers, and others) varied across the hospitals, ranging from 826 to 20,899 users per day in each hospital and on average, more than two (2.2 caregivers/patients) caregivers came to visit the hospital for patients care (Table 1).

Table 1: Descriptive statistics of selected hospitals (10 government and two non-government hospitals) in Dhaka, Bangladesh, July-December 2022.

Hospital type and codes	Year established	# of beds	# of patients[§]	# of caregivers and others[†]	# of staff	Total users[‡]	Total toilets	Overall: toilet to users ratio
**Govt. General hospitals**								
H1	2001	250	433	2571	472	3476	132	1:26
H2	2012	500	6746	12994	1159	20899	379	1:55
**Govt. Medical college hospitals**								
H3	1963	850	2612	6118	1548	10278	255	1:40
H4	2009	500	4703	11504	1150	17357	430	1:40
**Govt. specialized hospitals**								
Treat mainly infectious diseases								
H5	1972	300	503	1117	105	1725	43	1:40
H6	2018	250	663	679	597	1939	194	1:10
Treat mainly chronic diseases								
H7	1978	414	2002	5454	995	8451	318	1:27
H8	1982	300	2193	2965	715	5873	203	1:29
H9	2012	450	1779	3343	1562	6684	183	1:37
H10	2013	250	1011	2936	381	4328	177	1:24
**Private medical college hospitals**								
P1	1986	500	673	1089	378	2140	86	1:27
P2	1992	500	178	314	334	826	59	1:14
**Total/overall**	**-**	**5064**	**23496**	**51084**	**9396**	**83976**	**2459**	**1:34**

* The total number of inpatient and outpatient present/visited on the day of data collection
[†]Other users: security guard, canteen, and medical representatives
[‡]Total number of users including staff, patients, caregivers and other users use one toilet per day
"H"-represented the codes for government hospitals
"P"-represented the codes for private hospital

Among 2875 toilets, 86% (n=2459) were directly observed by the data collectors. The main reasons for not being able to observe (14%) toilets were either due to being locked (10%) or not obtaining permission to access the toilet. Among the toilets observed, we found 56% toilets were equipped with a sitting flush toilet while 44% featured a low pan/squat designed toilets. Almost all toilets (99%) had a functional water seal. The flooring material in nearly all toilets was comprised of ceramic tiles (98%). Additionally, the water supply to all toilets

was facilitated through a piped water system (Table 2). Overall, only 26% of the handwashing basins inside patient's toilets had soap for handwashing.

Table 2: Toilet observation status, overall toilet structure, functionality and water source for toilet use in 10 government hospitals and two non-government hospitals in Dhaka, Bangladesh, July-December 2022.

**Variables**	**Number n (%) N=2875**
**Able to observe toilet,** yes	2459 (86)
**Type of toilet pan**	
*Squatting pan flush toilet	1083 (44)
†Sitting pan flush toilet	1376 (56)
**Type of toilet floor**	
Ceramic tiles	2409 (98)
Cemented floor	30 (1)
Marble floor	19 (1)
**Type of toilet flush**	
Cistern/tank flush (functional)	1672 (68)
Cistern/tank flush (non-functional)	443 (18)
Pour/bucket flush	344 (14)
**Water seal,** Present	2439 (99)
**Water use option for personal cleaning**	
Tap water to bucket/water pot	1254 (51)
Hand shower	1156 (47)
Only tap water but no bucket	50 (2)
**Functional electric light present**	
Inside toilet block (Yes)	2066 (84)
Inside toilet cubicle (Yes)	1549 (63)
**Presence of function exhaust fan, yes**	197 (8)
**Toilet maintain privacy, yes**	2213 (90)
**Broken door (Yes)**	246 (10)
**Toilets with solid disposal bin (MHM facility)**	97 (3)
**Toilets for person with limited mobility (only handle)**	10 (0.3)

*A squat toilet (or squatting toilet) is a toilet used by squatting, rather than sitting. This means that the posture for defecation and for female urination is to place one foot on each side of the toilet drain or hole and to squat over it (Wikipedia: Squatting toilet).
†A typical flush toilet is a ceramic bowl (pan) connected on the "up" side to a cistern (tank) that enables rapid filling with water, and on the "down" side to a drain pipe that removes the effluent (Wikipedia: Squatting toilet).

The flushing mechanisms in the toilets varied, with 68% utilizing cistern/tank flush systems, of which 18% were non-functional. In contrast, 14% of the toilets were pour/bucket flush. For ~~toilet usage,~~ more than 51% of the facilities relied on both tap water and buckets, while 47% were equipped with a piped hand shower. Only 2% of the toilets provided tap water

without a bucket. None of the observed toilets provided toilet paper for patient use. Privacy of the users was adequately maintained across almost all toilets, although 10% of the toilet doors were found to be broken. Regarding facilities, we found that 84% of the toilet blocks had a minimum of one functional electric bulb, while only 8% had a functioning exhaust fan to maintain ventilation (Table 2).

We did not observe any signage indicating gender-segregated toilets for patient facilities. Both government and private hospitals exhibited a significant proportion of mixed-gender toilet usage. In government hospitals, 68% (n=629) of toilets were used by both male and female, and in private hospitals, this figure was 92% (n=97). Regarding inpatient wards, 68% (n=286) of users in government hospitals and 61% (n=17) of staff toilets were mixed-gender. We did not find any mixed-gender staff toilet in private hospitals (Table 3). Only 3% (n=97) of toilets had a bin for menstrual pad disposal and among them <1% bins had a functional lid. Overall, less than 1% (n=10) of the toilets had partial facilities (only handles) for individuals with limited mobility. Due to the absence of separate latrines designated for individuals with limited mobility, none of the selected hospitals met basic sanitary facilities according to JMP (Table 2).

Table 3: Descriptive statistics of health care facility, toilet users and ratio among 10 government hospitals and two non-government hospitals in Dhaka, Bangladesh, July-December 2022.

**Type of hospital (# of toilets)**	**[§]Functional toilets N/n (%)**			**[||]Clean toilets[a]**		
	**Overall**	**Inpatients departments**	**Outpatients department**	**Overall, n (%)**	**Inpatients departments (%)**	**Outpatients department (%)**
**Govt. general and medical college hospitals (n=1196)**	**823 (69)**	**926/629 (68)**	**270/194 (72)**	**359 (30)**	**307 (33)**	**52 (19)**
Male (n=141)	92 (65)	112/65 (58)	29/27 (93)	42 (30)	32 (29)	10 (34)
Female (n=129)	102 (79)	112/88 (79)	17/14 (82)	45 (35)	39 (35)	6 (35)
Mix-gender (n=926)	629 (68)	702/476 (68)	224/153 (68)	272 (29)	236 (34)	36 (16)
**[†]Staff (n=96)**	**58 (60)**	**92/54 (59)**	**4/4 (100)**	**13 (14)**	**13 (14)**	
Male (n=62)	35 (56)	61/34 (56)	1/1 (100)	6 (10)	6 (10)	
Female (n=6)	6 (100)	6/6 (100)	NA	2 (33)	2 (33)	**NA**
[‡] Mix-gender (n=28)	17 (61)	25/14 (56)	3/3 (100)	5 (18)	5 (20)	
***Patient's wards (n=487)**	**339 (70)**	**479/332 (69)**	**8/7 (88)**	**164 (34)**	**162 (34)**	**2 (25)**
Male patients (n=17)	14 (82)	17/14 (82)	NA	10 (59)	10 (59)	NA
Female patients (n=50)	39 (78)	50/39 (78)		15 (30)	15 (30)	
Mix-gender (n=420)	286 (68)	412/279 (68)	8/7 (88)	139 (33)	137 (33)	2 (25)
**Patient's service**	**256 (69)**	**347/235 (63)**	**26/21 (81)**	**133 (30)**	**132 (37)**	**1 (25)**
Cabins (n=82)	57 (70)	82/57 (70)	NA	52 (63)	52 (63)	
Special care (n=95)	80 (84)	90/76 (84)	5/4 (88)	16 (17)	16 (18)	NA
Pediatric and gynae (n=117)	71 (61)	114/71 (62)	3/0 (0)	43 (37)	43 (38)	
Emergency (n=10)	9 (90)	NA	10/9 (90)	0	NA	
Diagnostics/pharmacy/blood bank (n=31)	14 (45)	27/10 (37)	4/4 (100)	9 (29)	8 (30)	1 (25)
Others (n=38)	25 (66)	34/21 (62)	4/4 (100)	13 (34)	13 (38)	NA
**Govt. specialized hospitals (n=1118)**	**739 (66)**	**896/587 (66)**	**222/152 (68)**	**413 (37)**	**319 (36)**	**94 (42)**
Male (n=103)	74 (72)	81/57 (70)	22/17 (77)	57 (55)	46 (57)	11 (50)
Female (n=102)	76 (75)	79/61 (77)	23/15 (65)	66 (65)	51 (65)	15 (65)
Mix-gender (n=913)	589 (65)	736/469 (64)	177/120 (68)	290 (32)	222 (30)	68 (38)
**Staff and admin (112)**	**70 (63)**	**97/57 (59)**	**15/13 (87)**	**49 (44)**	**36 (37)**	**13 (87)**
Male (n=35)	21 (60)	32/19 (59)	3/2 (67)	18 (51)	16 (50)	2 (67)
Female (n=11)	9 (82)	8/7 (88)	3/2 (67)	9 (82)	7 (88)	2 (67)
Mix-gender (n=66)	40 (61)	57/31 (54)	9/9 (100)	22 (33)	13 (23)	9 (100)
**Patient's wards (n=597)**	**359 (60)**	**561/338 (60)**	**36/21 (58)**	**174 (23)**	**168 (30)**	**8 (22)**
Male (n= 34)	20 (59)	31/20 (65)	3/0 (0)	16 (47)	16 (52)	NA

Female (n=52)	34 (65)	49/33 (67)	3/1 (33)	28 (54)	27 (55)	1 (33)
Mix-gender (n=511)	305 (60)	481/285 (59)	30/20 (67)	132 (26)	125 (26)	7 (23)
**Patient's service (n=336)**	**264 (79)**	**230/186 (86)**	**106/78 (74)**	**168 (52)**	**111 (57)**	**57 (53)**
Cabins (n=45)	36 (80)	45/36 (80)	NA	30 (67)	30 (67)	NA
Special care (n=71)	55 (77)	71/55 (77)	NA	29 (41)	29 (41)	
Pediatric and gynae (n=15)	12 (80)	5/5 (100)	10/7 (70)	8 (53)	4 (80)	4 (40)
Emergency (n=19)	17 (89)	3/3 (100)	16/14 (88)	8 (42)	NA	8 (50)
Diagnostics/pharmacy/blood bank (n=161)	126 (78)	90/75 (83)	71/51 (72)	78 (48)	39 (43)	39 (55)
Other (n=25)	18 (72)	16/12 (75)	9/6 (67)	15 (60)	9 (56)	6 (67)
**Private hospitals (n=145)**	**134 (92)**	**123/114 (93)**	**22/20 (91)**	**81 (56)**	**70 (57)**	**11 (50)**
Male (n=7)	7 (100)	NA	7/7 (100)	2 (29)	0	2 (29)
Female (n=32)	**30 (94)**	28/26 (93)	4/4 (100)	22 (69)	18 (64)	4 (100)
Mix-gender (n=106)	97 (92)	95/88 (93)	11/9 (82)	57 (54)	52 (55)	5 (45)
**Patient's wards (n=73)**	**69 (95)**	**73/69 (95)**		**49 (67)**	**49 (67)**	
Female (n=14)	14 (100)	14/14 (100)	NA	12 (86)	12 (86)	NA
Mix-gender (n=59)	55 (93)	59/55 (93)		37 (63)	37 (63)	
**Patients service (n=53)**	**46 (87)**	**50/45 (93)**	**3/1 (33)**	**24 (51)**	**21 (48)**	
Special care (n=11)	11 (100)	11/11 (100)	NA	6 (55)	6 (55)	
Pediatric and gynae (n=25)	21 (84)	25/21 (84)		8 (32)	8 (32)	NA
Diagnostics/pharmacy/blood bank (n=8)	6 (75)	5/5 (100)	3/1 (33)	6 (75)	3 (60)	
Others (n=9)	8 (89)	9/8 (89)	NA	4 (44)	4 (44)	

*Patients users: Patients including caregivers of hospitals reported by hospital authority during the day of data collection

†Staff: toilet user's included doctors, nurse, and cleaning staff

‡Mix user: The toilet used by both patients, caregivers and staff

§Functional toilets: Toilet pan was not broken pan, functional door with lock, water available, not leakage of overhead sewage pipe, toilet pan was not overflowed or clogged

||Clean toilets: faeces were not visible on any surface of the toilet, no sputum/cough, solid waste, no insects and rodent were present, floor was not submerged with water, no strong smell of faeces

ɑThe denominator (N) for clean toilets are same as noted in the column under "Functional toilets" hence we did not repeat them.

The overall toilet-to-users ratio was substantially higher in the outpatient service than in the inpatient service (Fig 2). Specifically, in the outpatient service of government hospitals, the toilet-to-users ratio was 1:214, and in private hospitals, the ratio was 1:94. The highest toilet-to-users ratio was 1:450 in the outpatients of a government specialized hospital (H5; 300 beds), while the lowest ratio was 1:74 in the inpatients of a government general hospital (H6; 250 beds). In the outpatient service, the toilet-to-users ratio for mix (patient and staff) user toilets was high (1:183), with the highest ratio (1:939) observed in a government specialized hospital (H7; 414 beds). Conversely, the ratio was low for mixed (patient and staff) user toilets in the outpatient service of private hospitals (1:14). The overall toilet-to-users ratio for staff was lower in both government and private hospitals compared to the toilet-to-user ratio for patients (Fig 2).

Fig 2: Average number of users per toilet in different patient service and in different types of hospitals in Dhaka city.

In the inpatient service of government hospitals, the toilet-to-users ratio was 1:17, and in private hospitals, the ratio was 1:9. The highest toilet-to-users ratio was 1:36 in the inpatients of a government medical college hospital (H3; 850 beds), while the lowest ratio was 1:4 in the inpatients of a government general hospital (H1; 250 beds). The toilet-to-users ratio was similar for mix (patient and staff) user toilets in the inpatient service for both government (1:19) and private hospitals (1:18). The average toilet-to-users ratio in the inpatient service was also similar for both government (1:12) and private hospitals (1:13). The highest toilet-to-user ratio for staff was 1:70 in the inpatients of a government specialized hospital (H8; 300 beds), while the lowest ratio was 1:5 in the inpatients of both a

government general hospital (H2; 500 beds) and a government specialized hospital (H10; 250 beds). Similar results were observed in the toilet-to-users ratio for staff in the outpatient services of government and private hospitals. Six inpatient service toilets and four outpatient service toilets in government hospitals had fewer than 10 users per toilet for staff (Table 4). Toilet-to-users ratio further increased when we considered only functional toilets in the analysis (Table S1).

Table 4: Toilet to users' ratio, functionality, and cleanliness of toilets in 10 government hospitals and two non-government hospitals in Dhaka, Bangladesh, July-December 2022

	**Toilet-to-user ratio**						**[§]Functional toilets n (%)**		**[‖]Clean toilets n (%)**	
**Type of hospital (# of toilets)**	**Inpatient departments**			**Outpatient departments**			**Inpatient departments**	**Outpatient departments**	**Inpatient departments**	**Outpatient departments**
	**Toilet : patients[*]**	**Toilet : staff[†]**	**Toilet : mix[‡]**	**Toilet : patients**	**Toilet : staff**	**Toilet : mix**				
**Government hospitals (n=2314)**	**1:17**	**1:12**	**1:19**	**1:214**	**1:22**	**1:183**	**1216 (67)**	**346 (70)**	**626 (34)**	**146 (30)**
**General hospitals (n=511)**	**1:6**	**1:11**	**1:24**	**1:251**	**1:13**	**1:131**	**236 (64)**	**118 (81)**	**123 (34)**	**30 (20)**
H1(n=132)	1:4	1:36	1:51	0	1:11	1:42	61 (68)	27 (64)	23 (26)	0
H2 (379)	1:7	1:5	1:16	1:251	1:14	1:195	175 (63)	91 (88)	100 (36)	30 (29)
**Medical College hospitals (685)**	**1:20**	**1:10**	**1:15**	**1:172**	**1:68**	**1:264**	**393 (70)**	**76 (61)**	**184 (33)**	**22 (18)**
H3 (n=255)	1:36	1:14	1:23	1:175	1:14	1:197	154 (72)	28 (68)	56 (26)	5 (12)
H4 (n=430)	1:10	1:7	1:11	1:172	1:92	1:343	239 (69)	48 (57)	128 (37)	17 (20)
**Specialized hospitals (n=1118)**	**1:18**	**1:14**	**1:19**	**1:202**	**1:8**	**1:158**	**587 (66)**	**152 (68)**	**319 (36)**	**94 (42)**
Treat mainly infectious diseases										
H5 (n=43)	1:12	1:8	0	1:450	1:3	0	27 (69)	4 (100)	10 (26)	1 (25)
H6 (n=194)	1:10	1:7	1:15	1:74	1:4	0	106 (65)	16 (50)	76 (47)	11 (34)
Treat mainly chronic diseases										
H7 (n=318)	1:25	1:8	1:21	1:375	1:13	1:939	191 (64)	8 (42)	60 (20)	7 (37)
H8 (n=203)	1:23	1:70	1:21	1:114	1:7	1:13	56 (56)	63 (61)	21 (21)	48 (47)
H9 (n=183)	1:14	1:13	1:41	1:264	1:22	1:48	113 (80)	39 (95)	74 (52)	19 (46)
H10 (n=177)	1:31	1:5	1:14	1:101	1:3	1:368	94 (61)	22 (96)	78 (51)	8 (35)
**Private hospitals (n=145)**	**1:9**	**1:13**	**1:18**	**1:94**	**1:32**	**1:14**	**114 (93)**	**20 (91)**	**70 (57)**	**11 (50)**
P1 (n=86)	1:11	1:12	1:13	1:172	1:37	1:30	72 (99)	11 (85)	54 (74)	7 (54)
P2 (n=59)	1:3	1:16	1:21	0	1:5	1:4	42 (84)	9 (100)	16 (32)	4 (44)
**Overall total (n=2459)**	**1:17**	**1:12**	**1:19**	**1:205**	**1:22**	**1:176**	**1330 (68)**	**366 (71)**	**696 (36)**	**157 (31)**

[*]Patients users: Patients including caregivers of hospitals reported by hospital authority during the day of data collection
[†]Staff: toilet user's included doctors, nurse, and cleaning staff
[‡]Mix user: The toilet used by both patients, caregivers and staff
[§]Functional toilets: Toilet pan was not broken pan, functional door with lock, water available, not leakage of overhead sewage pipe, toilet pan was not overflowed or clogged
[‖]Clean toilets: faeces were not visible on any surface of the toilet, no sputum/cough, solid waste, no insects and rodent were present, floor was not submerged with water, no strong smell of faeces

Overall, 69% (n=1696) of toilets were found to be functional. The highest functionality rate was observed in female toilets compared to mixed users' toilets. Among staff toilets, mixed users' toilets exhibited the lowest level of functionality in comparison to male and female staff toilets. (Fig 3). In private hospitals, 100% of toilets were found to be functional in mixed-gender patient's ward toilets, and special care toilets. In government hospitals, only female staff toilets in general hospitals and medical college hospitals were 100% functional. The lowest toilet functionality was found in the diagnostics/pharmacy/blood bank areas of government hospitals (45%), followed by male staff (56%) and male patient toilets (59%) in specialized hospitals. In government general and medical college hospitals, 69% (n=823) of toilets were functional, with inpatient toilets showing 68% functionality and outpatient toilets demonstrating 72% functionality. Toilet functionality was greater (92%) in private hospitals (93% functionality for inpatient and 91% for outpatient toilets) compared to government hospitals (67% functionality for inpatient and 70% for outpatient toilets) (Table 3).

Fig 3: Functionality and cleanliness status of the toilets in healthcare facilities in Dhaka city according to gender and patients service.

Overall, fewer than 35% (n=853) toilets were observed to be clean. The highest level of cleanliness was observed in female toilets when compared to mixed users' toilets. Among staff toilets, mixed users' toilets showed the lowest level of cleanliness in comparison to male and female staff toilets. (Fig 3). Private hospitals exhibited the highest cleanliness rates, with 100% (n=9) cleanliness in mixed user staff and female toilets located outdoors. This was followed by 88% (n=7) cleanliness in female staff outpatients' facilities and 82% (n=9) in female staff inpatients' facilities. In government hospitals, the highest cleanliness rate, 63% (n=52), was in patients' personal cabins. Conversely, in government hospitals, no clean toilets

were found in emergency services, and only 16% of toilets were clean in mixed users patients' facilities. Cleanliness rates were 23% in patients' mixed user outpatients' toilets and 25% in the diagnostics/pharmacy/blood bank areas of government hospitals (Table 3). The perceived cleanliness of the toilets by the data collectors during toilet observation indicated that only 30% of toilets were reported as "not dirty at all," while more than 43% were reported as "little dirty," and the remaining 27% were described as quite dirty or unusable (Fig 4).

Fig 4: Cleanliness of toilets according to data collector’s perception during observation.

Our data reveals significant differences in toilet functionality and cleanliness across various parameters (Table 5). Private hospitals exhibited significantly higher rates of functional ($OR=3.73$, $p<0.001$) and clean toilets ($OR=8.49$, $p<0.001$) than government hospitals. Similarly, hospitals constructed after 2000 demonstrated significantly higher functionality and cleanliness compared to those built before 2000. Interestingly, toilets used by more than 30 users showed lower functionality rates ($OR=0.71$, $p<0.005$) and significantly lower cleanliness rates ($OR=0.44$, $p<0.001$) than those with fewer than 30 users. Staff-used toilets displayed significantly high functional ($OR=1.66$, $p<0.001$) and clean toilets ($OR=2.28$, $p<0.001$) than patient-used toilets. While toilets for females had higher functionality ($OR=1.36$, $p=0.132$), no difference in cleanliness was observed ($OR=1.43$, $p=0.057$) compared to male toilets. Additionally, no significant difference in functionality and cleanliness were observed between inpatient and outpatient services (Table 5).

Table 5: Toilet functionality and cleanliness of selected hospitals in 10 government hospitals and two non-government hospitals in Dhaka, Bangladesh, July-December 2022

**Hospital type (# of toilet)**	**Functional toilets, n**	**%**	**(OR, p-value)***	**Clean toilets, n**	**%**	**(OR, p-value)***
Government (N=2314)	1562	77.50		772	33.36	
Private (N=144)	134	92.41	(3.73, p<0.001)	81	55.85	(8.49, p<0.001)
**Year constructed**						
Before 2000 (N=714)	513	71.83		247	34.56	
After 2000 (N=1750)	1311	75.00	(1.46, p<0.001)	540	36.57	(1.24, p<0.05)
**Type of users**						
Patient’s toilet (N=871)	606	69.57		240	27.55	
Staff toilets (N=1054)	837	79.40	(1.66, p<0.001)	582	55.21	(2.28, p<0.001)
Mix user’s toilets (N=539)	381	70.70	(1.06, p=0.641)	119	22.08	(0.93, p=0.620)
**Patient’s service**						
Inpatients (N=1949)	1,413	72.50		721	37.00	
Outpatients (N=515)	411	79.81	(1.13, p=0.271)	166	32.25	(1.04, p= 0.734)
**Gender**						
Male (N=222)	169	76.12		106	47.75	
Female (N=276)	219	79.34	(1.36, p=0.132)	151	54.71	(1.43, p=0.057)
Mix-gender (N=1966)	1436	74.57	(1.05, p=0.765)	630	32.00	(0.89, p= 0.476)
**# of users per toilet**						
≤30 (N=1869)	1432	66.61		801	42.86	
>30 users (434)	302	69.60	(0.71, p<0.005)	54	12.44	(0.44, p<0.001)

*p value is generated by multiple logistic regression adjusting relevant co-variates

Discussion

This hospital-based study presents a comprehensive analysis of sanitation facilities in large HCFs in Dhaka, Bangladesh. It covers availability of sanitation facility, functionality, cleanliness, and toilet-to-user ratios in government and private hospitals within urban low-resource settings, as well as considering gender and inclusion aspects. The study also employed a novel methodology and data collection tools for precise toilet observation, accurately estimating toilet-to-user ratios among patient/caregiver, staff, and mixed users in complex HCFs. Moreover, this study included both government and private hospitals, covering various categories such as general, medical college, and specialized hospitals, thus ensuring the comprehensive representation of hospital types in Dhaka city. To our knowledge, there is a lack of studies estimating toilet conditions and appropriate toilet-to-user ratios in large urban hospitals in LMICs, which is important for Bangladesh due to higher patient loads in Dhaka compared to smaller cities [20, 32-34]. While previous studies in

Bangladesh and elsewhere in Africa and Asia assessed overall HCF WASH [32, 35-37], they rarely analyzed toilet-to-user ratio alongside functionality and cleanliness. A study assessed WASH in HCFs in Kampala, Uganda but lacked data on hospital size and daily patient load [36]. A nationwide Bangladesh study comprehensively analyzed HCF WASH, yet only reported toilet type, location, and overall cleanliness [32]. The paper categorized toilets using the 2015 WHO/UNICEF JMP classifications for improved toilets. Our current study provides comprehensive and detailed exploration of sanitation facilities in large HCFs within an urban low-resource setting. The findings and insights gained from this study provide important new evidence for policymakers, healthcare administrators and other researchers working in this field.

We consistently observed a toilet-to-users ratio in excess of 10 in outpatient services and in excess of 100 for outpatient services across the selected hospitals in Dhaka. At its most extreme, a toilet-to-user ratio of 1:939 was observed in an outpatient toilet within a government hospital. Similarly, both patient and mixed-user toilets consistently displayed increased ratio compared to staff toilets, whether in inpatient or outpatient services. Currently, there is a lack of data at local level on the appropriate toilet-to-users ratio in HCFs, including specific guidance for staff and patients. Furthermore, there is no information available regarding the implications of these high users ratios for public health, Infection Prevention and Control (IPC), and patient experience [20]. According to WaterAid's 2022 global technical guidelines for HCF toilets, achieving an appropriate toilet-to-user ratio involves having a dedicated toilet for women and men separately in each toilet block [26]. Additional toilets should be provided at a ratio of one per 20-25 patients/caregivers, with an extra toilet added for every 50 additional patients/caregivers [26]. For outpatient and caregiver services, maintaining a 2:1 ratio of female to male toilets

is recommended [38]. According to the Bangladeshi national WASH standard and implementation guidelines from 2021, hospitals are recommended to maintain a ratio of 1:6 (1 toilet for every 6 inpatient beds) to ensure sufficient sanitation facilities [39, 40]. The recent WHO-UNICEF report on Water and Sanitation for Health Facility Improvement Tool (WASH FIT) recommended having two or more improved toilets for outpatients, plus an additional toilet per 20 users/inpatients [21].

Our present toilet assessment indicates that while the toilet-to-users ratio aligned with the WaterAid and WASH FIT guidelines for staff and patients/caregiver's toilets in the inpatient service, it did not meet the required standard ratio for outpatient toilets and mix-user toilets in the inpatient service. Hospitals need to reassess their toilet infrastructure to match the actual patient loads, particularly in outpatient areas. To tackle the issue of high toilet-to-users ratio, adopting a multi-sectoral approach in healthcare facility design is crucial. As far back as 2008, other authors have called for collaborative efforts between healthcare, architecture, and urban planning sectors can result in well-designed building layouts that ensure toilets are distributed appropriately according to patient loads, while also incorporating gender, and disability-friendly facilities [41]. This comprehensive approach will ultimately lead to enhanced sanitation services and improved user experiences within healthcare facilities.

The functionality rates observed in this study were unacceptably high. Our findings indicated that approximately one-third of the toilets (31%) were non-functional, and two-thirds (65%) were unclean. The HCFs in Dhaka are already grappling with an excessively high number of toilet users. The prevalence of broken and unclean toilets exacerbates this situation, rendering it challenging and impractical to ensure safe sanitation services for both

patients and staff. There is scarce data regarding toilet functionality and cleanliness, particularly in large cities in LMICs [2]. While the Joint Monitoring Programme (JMP) offers a clear definition for toilet functionality, which includes factors like a toilet not being broken, the toilet pit not being blocked, and availability of water for flush/pour-flush toilets [1], the definition of a clean toilet for HCFs is not adequately outlined. Although the WHO's guide on environmental cleaning and IPC in HCFs in LMICs provides guidelines on how to clean toilets, it does not explicitly define clean and unclean toilets [14]. Notably, SNV's recent sanitation impact indicators and assessment tools for Bangladeshi HCFs includes toilet cleanliness as a success indicator to define hygienic sanitation facilities in such settings [34]. It is important to emphasize that toilet hygiene and cleanliness are closely intertwined with overall environmental or surface cleaning in hospitals and consider toilet hygiene as a part of overall IPC in HCFs [14, 36].

Several studies have concluded that unhygienic toilets in HCFs can contribute to the transmission of various diseases [8-13]. Our findings revealed that approximately 70% of government-affiliated toilets were found to be unclean, with around 27% of these facilities being notably dirty and unusable. Recent research has shown that public hospitals in Dhaka often face overcrowding issues, surpassing their patient care facility capacities (such as the number of available beds, waiting areas, and space for attendants) [20]. This strain negatively impacts on sanitation facilities, including insufficient hand hygiene infrastructure and inadequate infection control measures. Moreover, reports suggest that major hospitals in Dhaka lack adequate cleaning staff to maintain the prescribed IPC protocols [42]. Taken together, these congested conditions coupled with limited WASH amenities may heighten the likelihood of transmitting infectious diseases, like enteric and respiratory diseases both

from patients to their caregivers and vice versa, as well as among asymptomatic caregivers and other patients [8, 43, 44].

Our findings also suggested that several factors were associated with lower levels of toilet cleanliness such as high toilet-to-users ratio (in cases where toilets are used by more than 30 users), the age of the toilet facilities (with older buildings showing lower cleanliness), and the usage of mixed-gender toilets as opposed to gender-segregated ones. Furthermore, our results revealed that government-run toilets were significantly less clean in comparison to those in private hospitals. Similarly, toilet cleanliness exhibited a significant difference between staff and patient users, highlighting that toilets designated for patients were notably less clean than those designated for staff. Although no previous study in HCFs has analyzed the status of toilet cleanliness and the factors responsible for unclean toilets, a community-based study conducted in Kampala slums, Uganda, concluded that a large number of users, infrequent cleaning, and lack of cooperation among household users contribute to unclean toilets [28]. A multisectoral collaborative approach is necessary to ensure appropriate toilet-to-users ratio for both staff and patients, modify infrastructure to accommodate more patient toilets, especially in outpatient services, and develop and adopt separate cleaning protocols for sanitation facilities, particularly in large hospitals, incorporate sanitation and cleanliness standards [20, 45].

The study found that gendered sanitation needs were not addressed. A significant number of mixed-gender toilets was evident across all hospitals, and there was an absence of signage designating gender-segregated toilets (male/female) in any of the hospital toilet blocks. This observation highlights that despite separate designs for male and female toilets, there was consistent overlapping of male and female patients in all hospitals. This situation

compromises the privacy of female patients, caregivers, and hospital staff. As noted in other studies, it is crucial to have separate toilet blocks for women due to the need for more space, privacy, and time during urination and defecation, especially considering menstruation [46]. Ensuring privacy and safety is also crucial to prevent any increase in the risk of violence against women and girls or any feelings of vulnerability among users. User perception also plays a significant role; if a facility is perceived as unsafe, it can discourage usage and drive individuals towards potentially less hygienic alternatives [47].

Gendered needs as regards MHM were also not met. We found that only 3% toilets had trash bin for menstrual pad and solid disposal and among them <1% bin had functional lid. The lack of MHM facilities in HCFs not only affects the physical health and hygiene of females but also has broader implications for gender equality, patient care, and the overall well-being of individuals using and working in healthcare settings [48, 49]. Furthermore, sewerage systems in hospital are designed to carry water and fecal matter; discarding solid waste such as menstrual pads and cloth in toilets can block sewage pipes and led to non-functional toilets [30]. This also pose challenges for sewage treatment plants and can have significant cost and human resource and dignity implications [50].

From an inclusion perspective, needs for person with limited mobility were also not met. Our data indicates that no hospital had a toilet facility for people with limited mobility. To achieve basic sanitation in HCFs, each section/toilet block (male and female) should ideally have at least one accessible toilet cubicle for individuals with disabilities that meets national or international standards [2]. Hospitals admit a wide range of people with limited mobility every day [51]. However, globally, there is a lack of data concerning the number, types, and severity of disabled individuals in HCFs, particularly in LMICs [51]. People with physical

disabilities face a higher risk of falls and accidents when there are no toilets designed specifically for them. The absence of disability-friendly toilets in each latrine block can make it difficult for individuals with disabilities to use the facilities independently [52]. This lack of accessible toilets can result in challenges, including dependency, stress, embarrassment, discomfort, and difficulties in self-care. The absence of suitable toilet options may lead individuals with disabilities to avoid healthcare facilities, causing delayed care and negative health outcomes [52]. Given that none of the hospitals in Dhaka city addressed these critical indicators, none of the selected hospitals met the criteria for basic sanitary facilities. Ensuring toilets with MHM facilities for women and dedicated toilets for individuals with limited mobility are two essential components for achieving the basic sanitation standards outlined by the Joint Monitoring Programme (JMP) in HCFs [53].

Although our study possessed significant strengths, it has some limitations. Firstly, we did not include private hospitals systematically, primarily focusing on government healthcare facilities. While this approach ensured representation of government hospitals in Dhaka city, it limited insights into the sanitation situation in private hospitals. The inclusion of only two hospitals did not adequately represent the diversity within private hospitals in Dhaka city. Future research should include a more comprehensive and representative sample of private hospitals to offer a holistic view. Secondly, we couldn't observe 14% of toilets either because they were locked or due to restricted access permissions. The results of this study might vary if we had included the unobserved toilets. Third, data were collected at a specific time point between June and December 2022, spanning from the post-monsoon to the winter season. We were unable to account for potential variations in patient flow during various seasons or disease outbreaks. Previous studies conducted in HCFs in Dhaka demonstrated substantial increases in patient flow during epidemics and diverse seasons, including the rise

in winter-borne diseases [54, 55] and outbreaks of vector-borne diseases [56]. To gain a more comprehensive understanding, it is crucial to monitor patient flows consistently throughout the year and evaluate the seasonal fluctuations in toilet usage, as this could have implications for toilet cleanliness and functionality. Finally, the study was conducted after the COVID-19 pandemic, a period during which several hospitals were shifting from treating COVID-19 cases (i.e., COVID dedicated hospitals) to focusing on general medical care (i.e., all general hospitals). This transition may have influenced patient and caregivers flow and, as a result, could have affected toilet usage patterns. Because of this transition, patient numbers might have been lower than usual in certain hospitals studied, especially in the inpatient services. Consequently, this could have resulted in higher toilet-to-users ratio than what was estimated in this study.

Conclusions and recommendations

This research found sanitation facilities in hospitals in Dhaka to be inadequate from a number of perspectives. The HCFs in Dhaka city are grappling with a significant influx of patients and caregivers utilizing the toilets, which has led to a strain on the existing sanitation systems. The toilets designated for patients in outpatient services are facing an exceptionally high user load, warranting immediate attention and intervention to alleviate this situation. The prevalence of non-functional and unclean toilets further exacerbates the sanitation challenges within these hospitals. Additionally, the lack of gender-segregated toilets and menstrual hygiene management (MHM) facilities and lack of toilets for individuals with limited mobility in all 12 hospitals underscores the critical gaps in sanitation infrastructure. Without addressing these challenges, the goal of achieving basic and safe sanitation facilities for patients of hospitals and HCFs in dense urban settings such as Dhaka, and particularly for women and individuals with limited mobility by 2030 appears to be unattainable. The

government should allocate resources for the appropriate design of sanitation facilities in HCFs that meet acceptable user ratios, ensuring an adequate number of sanitation workers and providing proper training on toilet hygiene and IPC to improve the sanitation facilities in HCFs. Multisectoral approaches and adequately funded plans are urgently needed to improve the availability, functionality, and cleanliness of toilets in hospitals in Bangladesh and LMICs more broadly. Doing so will be critical to protecting public health and accelerating efforts towards Sustainable Development Goal 6 (SDG6).

Acknowledgments

We would like to convey our gratitude to the Directorate General of Health Services (DGHS) under Bangladesh's Ministry of Health and Family Welfare (MoHFW) for providing approval and necessary support to conduct the research. We acknowledged the Director, Hospital and Clinic Sections, DGHS, for his strong leadership and continuous support in collecting samples and conducting the study in selected COVID-19 and non-CVID19 hospitals. We are also thankful to the Directors, doctors, and support staff of all selected hospitals for their permission and support during sample and data collection, interviewing, and effective implementation at the facility. We are also indebted to icddr,b fieldworkers workers who contributed immensely to conducting field activities to make this study successful. Finally, we sincerely thank the Bill & Melinda Gates foundation (BMGF) for the funding support and technical collaboration to achieve the study goal. icddr,b is also grateful to the governments of Bangladesh and Canada, for providing core/unrestricted support.

Funding statement

This project has been funded by the Bill & Melinda Gates foundation (BMGF) [grant number INV-045360].

Declaration of competing interest

The authors declare that they have no known competing financial interests or personal relationships that could have appeared to influence the work reported in this paper.

CRediT Authorship contribution statement

Nuhu Amin: Conceptualization, Methodology, Validation, Formal analysis, Data curation, Writing – original draft, Review & editing, Visualization, Supervision. **Md. Imam Hossain:** Data collection, Methodology, Formal analysis, Writing – review & editing. **Rezaul Hasan:** Securing of Government approval, Writing – review & editing. **Supriya Sarker:** Securing of Government approval, Writing – review & editing. **Aninda Rahman:** Securing of Government approval, Writing – review & editing. **Mahbubur Rahman:** Conceptualization, Methodology, Investigation, Project Administration, Writing – review & editing. **Tim Foster:** Conceptualization, Methodology, Investigation, validation, Writing – supervision, review & editing. **Juliet WIlletts:** Conceptualization, Methodology, Investigation, validation, Writing – supervision, review & editing.

References:

[illegible] WHO. World Health Organization (2023). Water, sanitation, hygiene, waste and electricity services in health care facilities: progress on the fundamentals. 2023 Global Report. 2023.

2. WHO-UNICEF. Water, sanitation, hygiene, waste and electricity services in health care facilities: progress on the fundamentals.

2023 global report. Geneva: World Health Organization and the United Nations Children’s Fund (UNICEF), 2023. Licence: CC BY-NC-SA 3.0 IGO. 2023.

3. Bouzid M, Cumming O, Hunter PR. What is the impact of water sanitation and hygiene in healthcare facilities on care seeking behaviour and patient satisfaction? A systematic review of the evidence from low-income and middle-income countries. BMJ Glob

Health. 2018;3(3):e000648. Epub 2018/05/17. doi: 10.1136/bmjgh-2017-000648. PubMed PMID: 29765776; PubMed Central PMCID: PMCPMC5950627.

4. Kruk ME, Gage AD, Arsenault C, Jordan K, Leslie HH, Roder-DeWan S, et al. High-quality health systems in the Sustainable Development Goals era: time for a revolution. Lancet Glob Health. 2018;6(11):e1196-e252. Epub 2018/09/10. doi: 10.1016/s2214-109x(18)30386-3. PubMed PMID: 30196093; PubMed Central PMCID: PMCPMC7734391.

5. WHO. Water, sanitation and hygiene in health care facilities: status in low and middle income countries and way forward. Available at https://apps.who.int/iris/bitstream/handle/10665/154588/9789241508476_eng.pdf and accessed on May 22, 2023. 2015.

6. WHO-UNICEF. JMP WASH in Health care facilities. Available at https://washdata.org/monitoring/health-care-facilities and accessed on May 22, 2023. 2019.

7. WHO-UNICEF. Global progress report on WASH in health care facilities: Fundamentals first. Available at https://www.who.int/publications/i/item/9789240017542 and accessed on May 22, 2023. 2021.

8. Abney SE, Bright KR, McKinney J, Ijaz MK, Gerba CP. Toilet hygiene-review and research needs. J Appl Microbiol. 2021;131(6):2705-14. Epub 2021/04/27. doi: 10.1111/jam.15121. PubMed PMID: 33899991; PubMed Central PMCID: PMCPMC9292268.

9. Couturier J, Ginevra C, Nesa D, Adam M, Gouot C, Descours G, et al. Transmission of Legionnaires' Disease through Toilet Flushing. Emerg Infect Dis. 2020;26(7):1526-8. Epub 2020/06/23. doi: 10.3201/eid2607.190941. PubMed PMID: 32568063; PubMed Central PMCID: PMCPMC7323554.

10. Ding Z, Qian H, Xu B, Huang Y, Miao T, Yen HL, et al. Toilets dominate environmental detection of severe acute respiratory syndrome coronavirus 2 in a hospital. Sci Total Environ. 2021;753:141710. Epub 2020/09/07. doi: 10.1016/j.scitotenv.2020.141710. PubMed PMID: 32891988; PubMed Central PMCID: PMCPMC7428758.

11. Korpela J, Kärpänoja P, Taipalinen R, Siitonen A. Subtyping of Shigella sonnei for tracing nosocomial transmission. J Hosp Infect. 1995;30(4):261-6. Epub 1995/08/01. doi: 10.1016/0195-6701(95)90260-0. PubMed PMID: 7499806.

12. Knowlton SD, Boles CL, Perencevich EN, Diekema DJ, Nonnenmann MW, Program CDCE. Bioaerosol concentrations generated from toilet flushing in a hospital-based patient care setting. Antimicrobial Resistance & Infection Control. 2018;7(1):16. doi: 10.1186/s13756-018-0301-9.

13. Sun S, Han J. Open defecation and squat toilets, an overlooked risk of fecal transmission of COVID-19 and other pathogens in developing communities. Environmental Chemistry Letters. 2021;19(2):787-95. doi: 10.1007/s10311-020-01143-1.

14. WHO. Environmental cleaning and infection prevention and control in health care facilities in low- and middle-income countries: trainer's guide. Geneva: World Health Organization; 2022. Licence: CC BY-NC-SA 3.0 IGO. 2023.

15. Dancer SJ. The role of environmental cleaning in the control of hospital-acquired infection. J Hosp Infect. 2009;73(4):378-85. Epub 2009/09/04. doi: 10.1016/j.jhin.2009.03.030. PubMed PMID: 19726106.

16. BBS. Bangladesh Bureau of Statistics. Population Census 2022. Available at https://bbs.gov.bd/ and accessed on August 22, 2023. 2023.

17. Andaleeb SS, Siddiqui N, Khandakar S. Patient satisfaction with health services in Bangladesh. Health Policy and Planning. 2007;22(4):263-73. doi: 10.1093/heapol/czm017.

18. Kabir A, Karim MN, Billah B. Health system challenges and opportunities in organizing non-communicable diseases services delivery at primary healthcare level in Bangladesh: A qualitative study. Front Public Health. 2022;10:1015245. Epub 2022/11/29. doi: 10.3389/fpubh.2022.1015245. PubMed PMID: 36438215; PubMed Central PMCID: PMCPMC9682236.

19. Shahen MA, Islam MR, Ahmed R. Challenges for health care services in Bangladesh: an overview. IOSR Journal of Nursing and Health Science. 2020;9:13-24.

20. Amin N, Haque R, Rahman MZ, Rahman MZ, Mahmud ZH, Hasan R, et al. Dependency of sanitation infrastructure on the discharge of faecal coliform and SARS-CoV-2 viral RNA in wastewater from COVID and non-COVID hospitals in Dhaka, Bangladesh. Sci Total Environ. 2023;867:161424. Epub 2023/01/10. doi: 10.1016/j.scitotenv.2023.161424. PubMed PMID: 36623655; PubMed Central PMCID: PMCPMC9822545.

21. WHO-unicef. Water and Sanitation for Health Facility Improvement Tool (WASH FIT): A practical guide for improving quality of care through water, sanitation and hygiene in health care facilities. 2022.

22. WHO-WaterAid. DRAFT: Manual for Building Resilient Health Care Facilities in Climate Stressed Zones through Adaptive Technology and Effective WASH Facilities. 2022.

23. Berihun G, Adane M, Walle Z, Abebe M, Alemnew Y, Natnael T, et al. Access to and challenges in water, sanitation, and hygiene in healthcare facilities during the early phase of the COVID-19 pandemic in Ethiopia: A mixed-methods evaluation. PLOS ONE. 2022;17(5):e0268272. doi: 10.1371/journal.pone.0268272.

24. Grut L, Mji G, Braathen SH, Ingstad B. Accessing community health services: challenges faced by poor people with disabilities in a rural community in South Africa. Afr J Disabil. 2012;1(1):19. Epub 2012/10/10. doi: 10.4102/ajod.v1i1.19. PubMed PMID: 28729977; PubMed Central PMCID: PMCPMC5442570 may have inappropriately influenced them in writing this paper.

25. Boniol M, McIsaac M, Xu L, Wuliji T, Diallo K, Campbell J. Gender equity in the health workforce: analysis of 104 countries. Geneva: World Health Organization, 2019 2019. Report No.: Contract No.: WHO/HIS/HWF/Gender/WP1/2019.1.

26. WaterAid. Water, sanitation and hygiene in health care facilities: driving transformational change for women and girls. Ottawa: WaterAid Canada; 2019 (https://www.wateraid.org/ca/stories/watersanitationand-hygiene-in-health-care-facilities-driving-transformational-change-for, accessed 22 Aug 2023). 2019.

27. Duijster D, Monse B, Marquez M, Pakes U, Stauf N, Benzian H. Improving Toilet Usability and Cleanliness in Public Schools in the Philippines Using a Packaged Operation and Maintenance Intervention. International journal of environmental research and public health. 2022;19(16). Epub 20220815. doi: 10.3390/ijerph191610059. PubMed PMID: 36011699; PubMed Central PMCID: PMCPMC9407854.

28. Tumwebaze IK, Mosler H-J. Shared toilet users' collective cleaning and determinant factors in Kampala slums, Uganda. BMC public health. 2014;14(1):1260. doi: 10.1186/1471-2458-14-1260.

29. Talib YA, Abdul Ghani NI, Ismail K, Salleh NA. The Provision of the Disabled Facilities in Public Hospitals. MATEC Web Conf. 2016;66:00081.

30. Yeasmin F, Luby SP, Saxton RE, Nizame FA, Alam M-U, Dutta NC, et al. Piloting a low-cost hardware intervention to reduce improper disposal of solid waste in communal toilets in low-income settlements in Dhaka, Bangladesh. BMC Public Health. 2017;17(1):682. doi: 10.1186/s12889-017-4693-x.

31. Bursac Z, Gauss CH, Williams DK, Hosmer DW. Purposeful selection of variables in logistic regression. Source Code Biol Med. 2008;3:17. Epub 20081216. doi: 10.1186/1751-0473-3-17. PubMed PMID: 19087314; PubMed Central PMCID: PMCPMC2633005.

32. Unicomb L, Horng L, Alam MU, Halder AK, Shoab AK, Ghosh PK, et al. Health-Care Facility Water, Sanitation, and Health-Care Waste Management Basic Service Levels in Bangladesh: Results from a Nation-Wide Survey. Am J Trop Med Hyg. 2018;99(4):916-23. Epub 2018/08/29. doi: 10.4269/ajtmh.18-0133. PubMed PMID: 30152311; PubMed Central PMCID: PMCPMC6159562.

33. Horng LM, Unicomb L, Alam MU, Halder AK, Shoab AK, Ghosh PK, et al. Healthcare worker and family caregiver hand hygiene in Bangladeshi healthcare facilities: results from the Bangladesh National Hygiene Baseline Survey. Journal of Hospital Infection. 2016;94(3):286-94. doi: https://doi.org/10.1016/j.jhin.2016.08.016.

34. SNV. SNV in Bangladesh, Summary report. WASH in Health Care Facilities in Bangladesh: Policy review, stakeholders' mapping and baseline census of six urban centres, Dhaka. 2020.

35. Guo A, Bowling JM, Bartram J, Kayser G. Water, Sanitation, and Hygiene in Rural Health-Care Facilities: A Cross-Sectional Study in Ethiopia, Kenya, Mozambique, Rwanda, Uganda, and Zambia. Am J Trop Med Hyg. 2017;97(4):1033-42. Epub 2017/08/19. doi: 10.4269/ajtmh.17-0208. PubMed PMID: 28820718; PubMed Central PMCID: PMCPMC5637612.

36. Kayiwa D, Mugambe RK, Mselle JS, Isunju JB, Ssempebwa JC, Wafula ST, et al. Assessment of water, sanitation and hygiene service availability in healthcare facilities in the greater Kampala metropolitan area, Uganda. BMC Public Health. 2020;20(1):1767. doi: 10.1186/s12889-020-09895-9.

37. Odagiri M, Cahyorini, Azhar K, Cronin AA, Gressando Y, Hidayat I, et al. Water, Sanitation, and Hygiene Services in Public Health-Care Facilities in Indonesia: Adoption of World Health Organization/United Nations Children's Fund Service Ladders to National Data Sets for a Sustainable Development Goal Baseline Assessment. Am J Trop Med Hyg. 2018;99(2):546-51. Epub 2018/06/27. doi: 10.4269/ajtmh.18-0044. PubMed PMID: 29943724; PubMed Central PMCID: PMCPMC6090366.

38. WaterAid. Technical guidelines for construction of institutional and public toilets - Annexes. Available at https://washmatters.wateraid.org/sites/g/files/jkxoof256/files/annexes-to-technical-guidelines-for-construction-of-institutional-and-public-toilets.pdf and accessed on May 22, 2023. 2022.

39. WHO-IEDCR-WaterAid. DRAFT: Manual for Building Resilient Health Care Facilities in Climate Stressed Zones through Adaptive Technology and Effective WASH Facilities. 2022.

40. MoHFW. National Strategy for WASH in Health Care Facilities 2019-2023 A Framework
for Action. Available at. https://old.dghs.gov.bd/images/docs/Guideline/National %20Strategy%20for%20Wash%20in%20Healthcare%20Facilities%202019-2023.pdf. (Accessed 17 May 2022). 2019.

41. Chartier Y, Adams J, Bartram J. Essential environmental health standards in health care. Geneva: WHO. 2008.

42. WHO. Advancing on Infection Prevention and Control on World Patient Safety Day. Available at https://www.who.int/bangladesh/news/detail/20-09-2020-advancing-on-infection-prevention-and-control-on-world-patient-safety-day and accessed on August 27 2023. 2020.

43. Horng LM, Unicomb L, Alam MU, Halder AK, Shoab AK, Ghosh PK, et al. Healthcare worker and family caregiver hand hygiene in Bangladeshi healthcare facilities: results from the Bangladesh National Hygiene Baseline Survey. J Hosp Infect. 2016;94(3):286-94. Epub 2016/09/26. doi: 10.1016/j.jhin.2016.08.016. PubMed PMID: 27665311; PubMed Central PMCID: PMCPMC5495692.

44. Islam MS, Luby SP, Sultana R, Rimi NA, Zaman RU, Uddin M, et al. Family caregivers in public tertiary care hospitals in Bangladesh: risks and opportunities for infection control. Am J Infect Control. 2014;42(3):305-10. Epub 2014/01/11. doi: 10.1016/j.ajic.2013.09.012. PubMed PMID: 24406254; PubMed Central PMCID: PMCPMC4681270.

45. SNV. Summary report on WASH in Health Care Facilities in Bangladesh: Policy review, stakeholders' mapping and baseline census of six urban centres Available at

https://snv.org/cms/sites/default/files/explore/download/wash-in-healthcare-facilities-summary-report-urban-sanitation-bangladesh_0.pdf and Accessed 9th Sep 2021. 2020.

46. WaterAid-Unicef. Female-friendly public and community toilets: a guide for planners and decision makers. Available at https://washmatters.wateraid.org/sites/g/files/jkxoof256/files/female-friendly-public-and-community-toilets-a-guide.pdf and accessed on August 23, 2023.

47. Sharma DRP, B.; Pathak, R. P.; Shrestha, S. C. Healthcare liquid waste management. Journal of Nepal Health Research Council. 2010;8(1):23-6. Epub 2010/04/01. PubMed PMID: 21879009.

48. WaterAid-UNFPA. Menstrual Health and Hygiene Management for Persons with Disability:Insights and Good Practices from India. Available at https://www.wateraid.org/in/sites/g/files/jkxoof336/files/2022-05/Final%20MHHM%20-%20For%20Persons%20with%20Disability%20%281%29.pdf and accessed on August 25 2023. 2022.

49. WaterAid. Violence, gender and WASH toolkit-in Gender, Water, Sanitation, Hygiene. Available at https://washmatters.wateraid.org/publications/violence-gender-and-wash-toolkit and accessed on August 13, 2023. 2017.

50. Elledge MF, Muralidharan A, Parker A, Ravndal KT, Siddiqui M, Toolaram AP, et al. Menstrual Hygiene Management and Waste Disposal in Low and Middle Income Countries-A Review of the Literature. Int J Environ Res Public Health. 2018;15(11). Epub 2018/11/18. doi: 10.3390/ijerph15112562. PubMed PMID: 30445767; PubMed Central PMCID: PMCPMC6266558.

51. Morris MA, Wong AA, Dorsey Holliman B, Liesinger J, Griffin JM. Perspectives of Patients with Diverse Disabilities Regarding Healthcare Accommodations to Promote Healthcare Equity: a Qualitative Study. J Gen Intern Med. 2021;36(8):2370-7. Epub 2021/02/11. doi: 10.1007/s11606-020-06582-8. PubMed PMID: 33564941; PubMed Central PMCID: PMCPMC8342676.

52. McPherson A, Durham J, Richards N, Gouda H, Rampatige R, Whittaker M. Strengthening health information systems for disability-related rehabilitation in LMICs. Health Policy Plan. 2017;32(3):384-94. Epub 2016/12/10. doi: 10.1093/heapol/czw140. PubMed PMID: 27935799; PubMed Central PMCID: PMCPMC5400158.

53. WHO-UNICEF. JMP-WASH in Healthcare facility. 2022.

54. Hasan SMT, Das S, Faruque ASG, Khan AI, Clemens JD, Ahmed T. Taking care of a diarrhea epidemic in an urban hospital in Bangladesh: Appraisal of putative causes, presentation, management, and deaths averted. PLoS Negl Trop Dis. 2021;15(11):e0009953. Epub 20211115. doi: 10.1371/journal.pntd.0009953. PubMed PMID: 34780462; PubMed Central PMCID: PMCPMC8629377.

55. Schwartz BS, Harris JB, Khan AI, Larocque RC, Sack DA, Malek MA, et al. Diarrheal epidemics in Dhaka, Bangladesh, during three consecutive floods: 1988, 1998, and

2004. Am J Trop Med Hyg. 2006;74(6):1067-73. PubMed PMID: 16760521; PubMed Central PMCID: PMCPMC1626162.

56. Haider N, Chang YM, Rahman M, Zumla A, Kock RA. Dengue outbreaks in Bangladesh: Historic epidemic patterns suggest earlier mosquito control intervention in the transmission season could reduce the monthly growth factor and extent of epidemics. Curr Res Parasitol Vector Borne Dis. 2021;1:100063. Epub 20211126. doi: 10.1016/j.crpvbd.2021.100063. PubMed PMID: 35284868; PubMed Central PMCID: PMCPMC8906128.

Supporting information captions

Table S1: Toilet to users' ratio in 10 government hospitals and two non-government hospitals in Dhaka city, considering only functional toilets.

Fig S1: Perceived toilet cleanliness scale of the data collectors during observation

Figure

Click here to access/download;Figure;Fig 1.png

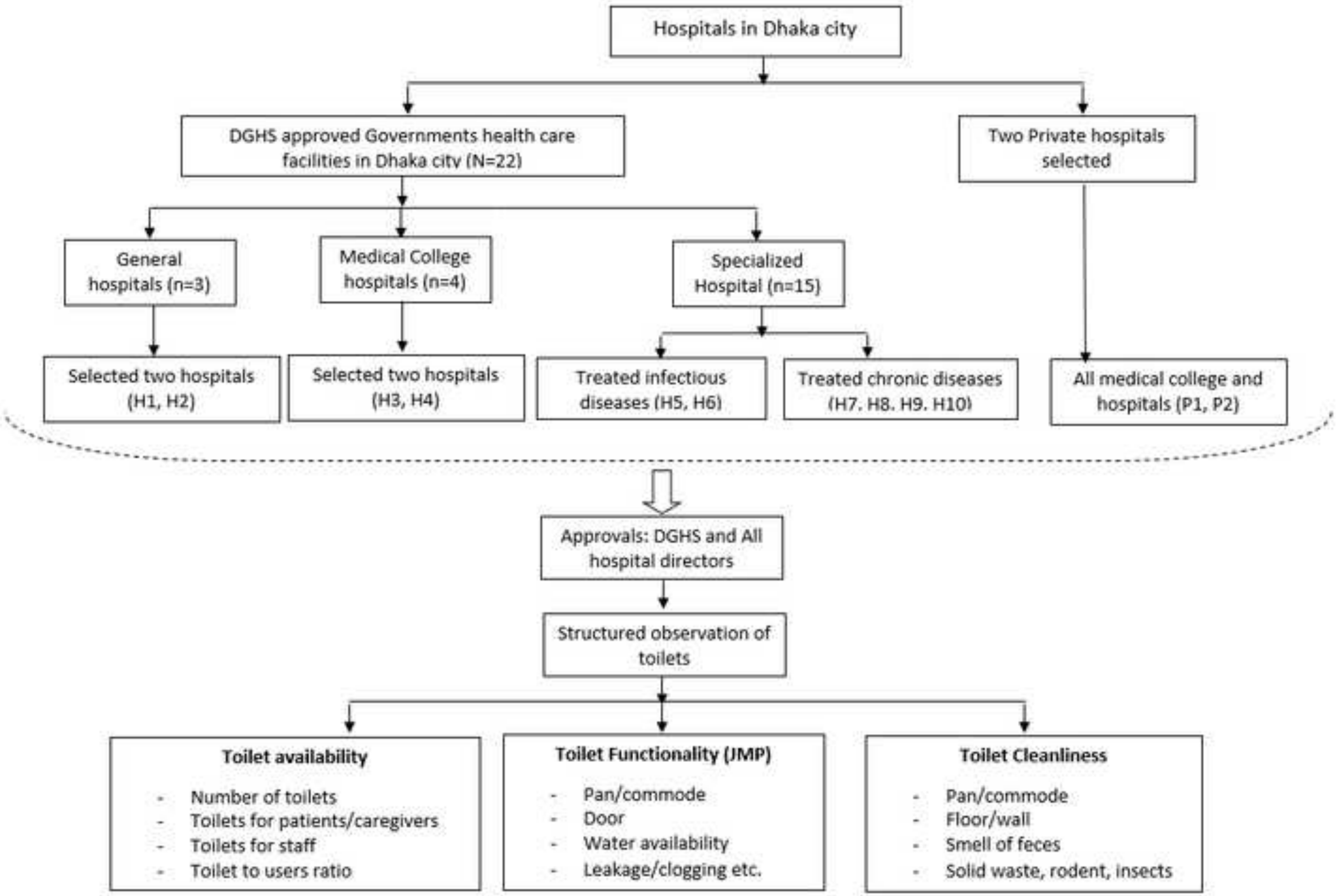

Figure

Click here to access/download;Figure;Fig 2.tif

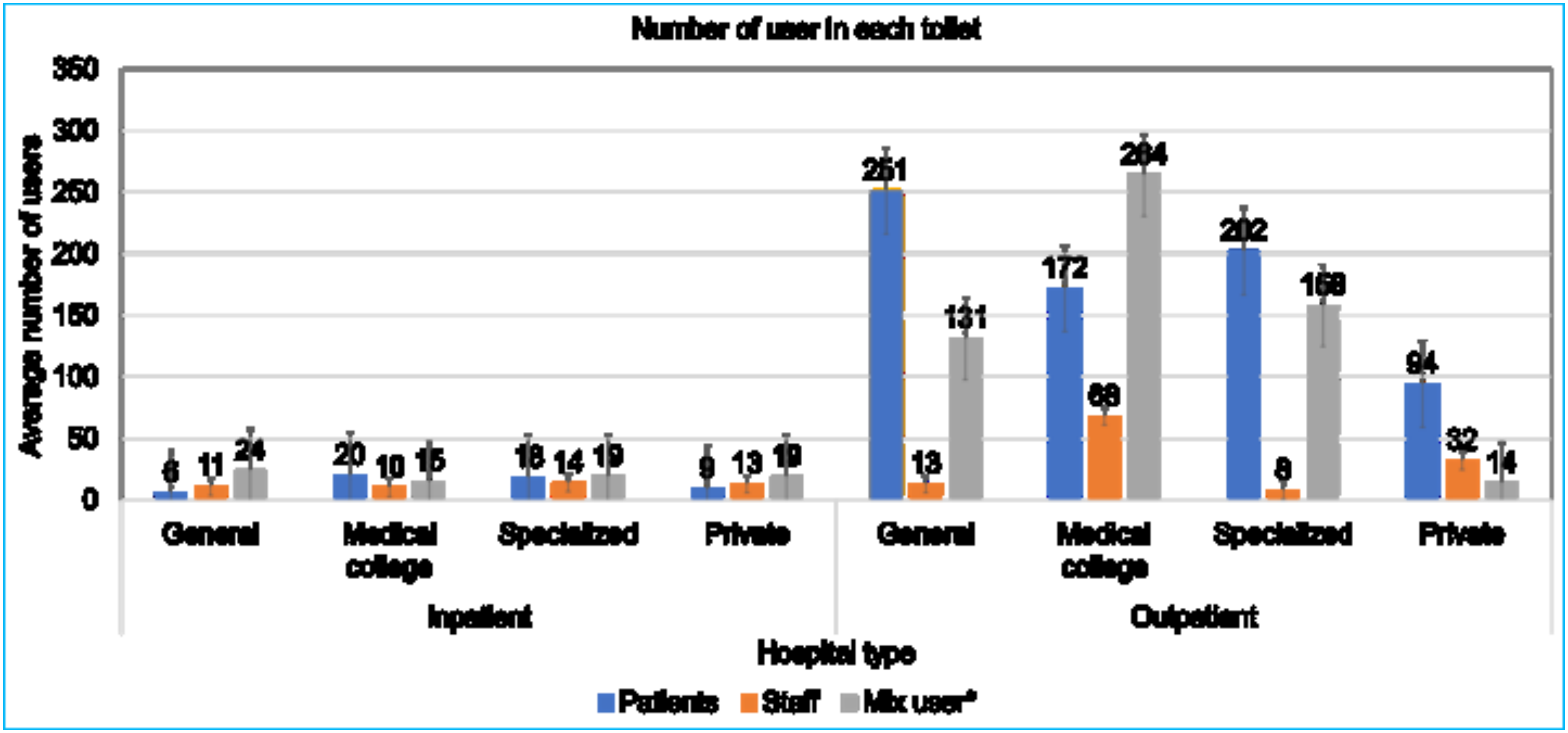

Figure

Click here to access/download;Figure;Fig 3.PNG

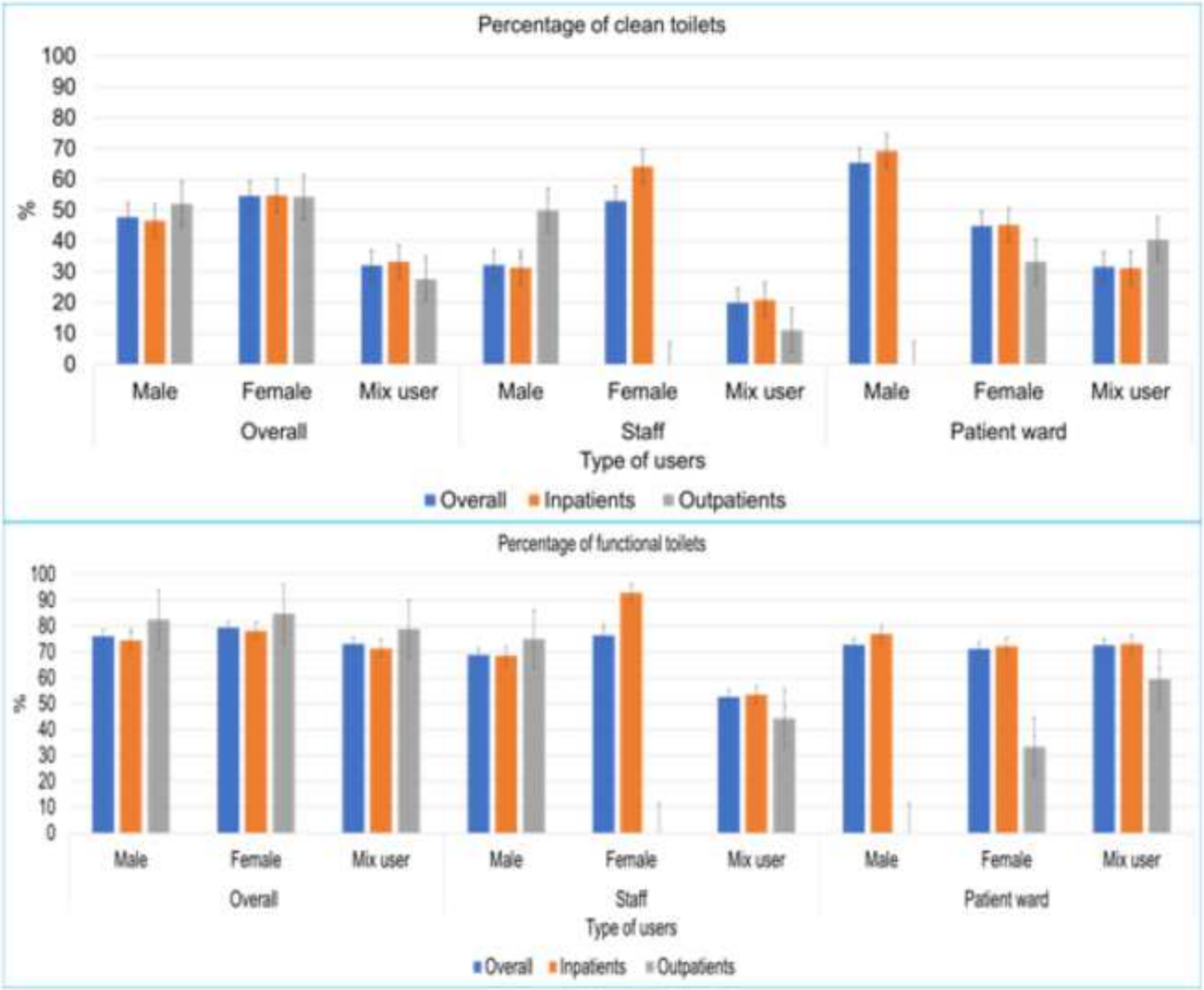

Figure Click here to access/download;Figure;Fig 4.tif

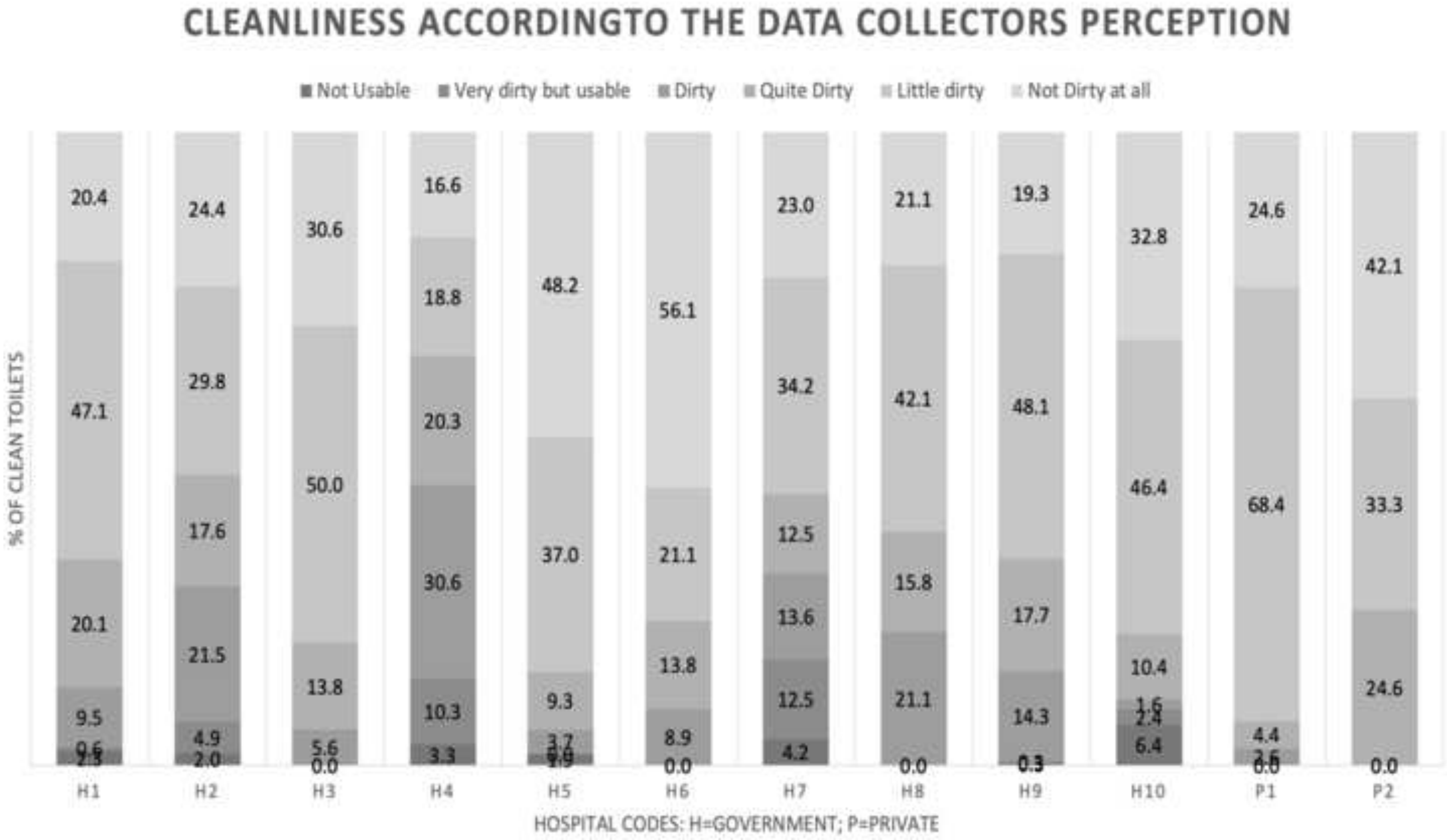

Click here to access/download;Figure;Fig S1.tif

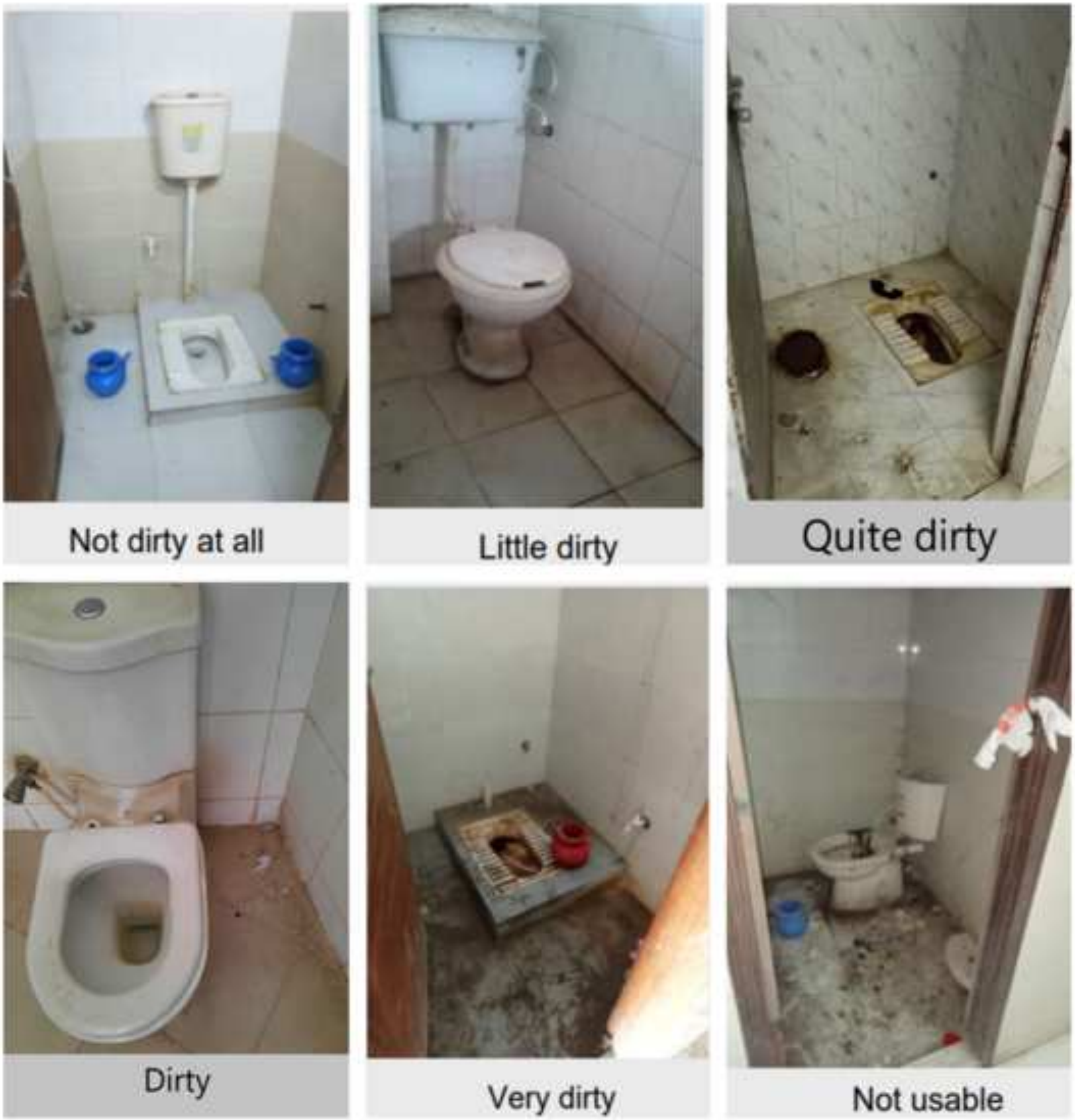

Click here to access/download
Supporting Information
Table S1.docx
