# Supplementary material for: Inadequate sanitation in healthcare facilities: A comprehensive evaluation of toilets in major hospitals in Dhaka, Bangladesh
Source: PLoS One. 2024 May 22;19(5):e0295879. doi: 10.1371/journal.pone.0295879 (PMC11111017; doi:10.1371/journal.pone.0295879)
Supplement: Attachment — Submitted filename: Response to the Reviewers.docx [file pone.0295879.s003.docx]

**Response to the reviewers’ comments:**

Dear Reviewer,

Thank you for providing additional feedback on the manuscript titled “*Inadequate Sanitation in Healthcare Facilities: A Comprehensive Evaluation of Toilets in Major Hospitals in Dhaka, Bangladesh.*” Your suggestions have significantly improved the clarity of the manuscript. We have carefully reviewed the comments and addressed them accordingly. Please find our responses in both track change format and clean version to the specific queries below:

**Review Comments to the Author**

**Reviewer #3**: Congratulations to the authors for addressing all the comments. I find the paper much improved, and am happy to recommend it be accepted.

**Response:** Much appreciate your time and efforts providing valuable suggestions to improve the manuscript.
I have a few small suggests for final edits:

-the JMP is mentioned in line 68 with the word "also", but the JMP has not been introduced in the text at this point in the paper.

**Response:** Removed “also” from the sentence.

-I would be careful about suggesting that the JMP "recommends" anything (line 99) - the JMP has definitions and indicators, but is itself not a normative agency

**Response:** Thinks for the suggestion. I have removed the JMP recommendation and added WaterAid users-to-toilet information.

-line 101 mentions WHO-JMP but this is not correct - I think you mean WHO-UNICEF JMP

**Response:** Thanks again for your careful review. We have corrected accordingly.

-the text on lines 103-107 is in the wrong place - should be in a separate section on study limitations or with other text describing the study methodology

**Response:** We have removed the section.

-on line 178 I think the text is supposed to say "we adopted....the JMP definitions..." the word definitions is missing

**Response:** Corrected accordingly.

-WASH FIT is mentioned in line 180 but has not been described - it is introduced much later in the paper -not until line

**Response:** We have elaborate/define the WASH-FIT.

-line 364 mentions that <1% of bins had a functional lid - this is such a small percentage that perhaps it makes sense to mention the exact number (I assume in single digits!)

**Response:** added (n=8)

-I not that for some of the tables the edit I suggested, having the percentage first and the n number in parentheses, has been adopted, but not in Table 4 or Table 5. Up to the editor to direct you, but I would be inclined to fix this inconsistency

**Response:** I would like to keep the number first and then the percentage [n (%)]. Both of the style is acceptable globally including PlosOne policy.

-not clear why you used a 2015 JMP report for classifications - this is the end of the MDG era

**Response:** We have used 2019 classification from WASH data. We corrected the year and put citation.

- perhaps you need to make this clear - that these are not the same classifications that are used currently by JMP

**Response:** I think 2015 was a typo. We used latest classification by JMP WASH data (washdata.org/monitoring/sanitation)

-line 521 says there is a lack of "data" on the appropriate user to toilet ratio but of course data don't tell you what is appropriate or not...pehraps you mean there is a lack of "guidance" on the appropriate ratio?

**Response:** Corrected the wording and used “guidance”

-line 537 says the "user to toilet ratio aligned" but do you mean the average ratio across all HCF studied? if so, specify...otherwise this is vague

**Response:** Thanks for your suggestion. You are right this was the average of all HCFs. Corrected accordingly,

“Our present toilet assessment indicates that while the average user-to-toilet ratio across all HCFs aligned with the WHO WASH FIT”

-line 541 -data "suggest"? shouldn't this be "showed" or "revealed"?

**Response:** Corrected and used “revealed”.

-line 591 is awkward - "toilet cleanliness exhibited"?

**Response:** Corrected accordingly.

“Our results also revealed that toilets designated for patients were notably less clean than those designated for staff.”

-I don't think the Uganda example mentioned in line 595 is relevant - not HCF toilets (so cooperation among households not relevant)

**Response:** We agree with you and removed the sentence accordingly.

-line 624 seems to suggest problems at sewage treatment plants have an impact on dignity - the reference to dignity does not belong here - maybe in line 619?

**Response:** Thanks for the suggestion. We have revised accordingly.
